# Serotonin Signals Overcome Loser Mentality in *Drosophila*

**DOI:** 10.1016/j.isci.2020.101651

**Published:** 2020-10-06

**Authors:** Shao Wei Hu, Yan Tong Yang, Yuanjie Sun, Yin Peng Zhan, Yan Zhu

**Affiliations:** 1 State Key Laboratory of Brain and Cognitive Science, Institute of Biophysics, Chinese Academy of Sciences, 15 Datun Road, Beijing 100101, China; 2 University of Chinese Academy of Sciences, Beijing 100049, China; 3 Sino-Danish College, University of Chinese Academy of Sciences, Beijing, China; 4 Sino-Danish Center for Education and Research, Beijing 100190, China; 5 Advanced Innovation Center for Human Brain Protection, Capital Medical University, Beijing, China

**Keywords:** Behavioral Neuroscience, Molecular Neuroscience, Cellular Neuroscience

## Abstract

Traumatic experiences generate stressful neurological effects in the exposed persons and animals. Previous studies have demonstrated that in many species, including *Drosophila*, the defeated animal has a higher probability of losing subsequent fights. However, the neural basis of this “loser effect” is largely unknown. We herein report that elevated serotonin (5-HT) signaling helps a loser to overcome suppressive neurological states. Coerced activation of 5-HT neurons increases aggression in males and promotes losers to both vigorously re-engage in fights and even defeat the previous winners and regain mating motivation. P1 neurons act upstream and 5-HT1B neurons in the ellipsoid body act downstream of 5-HT neurons to arouse losers. Our results demonstrate an ancient neural mechanism of regulating depressive behavioral states after distressing events.

## Highlights

• Activating a small subset of serotonin neurons promotes losers to fight
• Serotonin is necessary and sufficient for modulating aggression in losers
• The neural circuit for motivating losers includes P1, 5-HT, and 5-HT1B neurons
• Elevating 5-HT signaling overcomes the depressive behavioral state in losers

## Graphical Abstract

**Figure:**
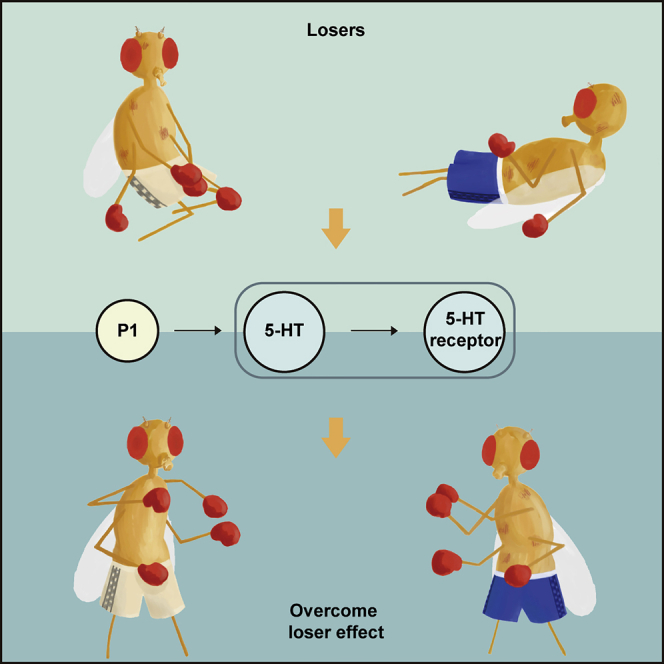

## Introduction

Aggression is widespread in the animal kingdom as an important form of social behavior. Fighting is crucial for defense against predators and competition for territory, food, or mates (Anholt and Mackay, 2012; Miczek et al., 2001; Packer et al., 1995; Sapolsky, 2005; Zwarts et al., 2012). Among social animals, aggressive displays serve to establish social hierarchy (Anholt and Mackay, 2012). As stressful and sometimes life-threatening experiences, aggressive encounters have long-lasting effects and change the mental states of the animals (Ehlers and Clark, 2000; Hofmann and Stevenson, 2000; Hsu and Wolf, 1999; Rutte et al., 2006). The development of relevant behavioral models is therefore needed to study behavior modulation and understand the mechanisms of how stressful experiences generate scarring neurological effects on the exposed persons and animals, such as those with post-traumatic stress disorder (Siegmund and Wotjak, 2006).

The consequence of aggressive rivalry has profound effects on decisions regarding “fight or flight” in subsequent social encounters. For example, mice exhibit a “winner effect” in which prior winning increases aggression and the probability of subsequent victory. The synaptic strength in the mediodorsal thalamus-dorsomedial prefrontal cortex circuit was reported to underlie the winner effect in mice (Zhou et al., 2017). Similarly, a “loser effect” is common in many animals, in which a prior losing experience decreases the probability of an individual winning a subsequent fight (Hsu and Wolf, 1999; Rutte et al., 2006). For example, a resident hamster normally displays territorial aggression to an intruding hamster, but after repeated defeats, the resident hamster behaves defensively and flees from an intruder (Hebert et al., 1996). In general, an individual with losing experiences is less likely to initiate a confrontation and exhibits an increased tendency to retreat when challenged (Hsu and Wolf, 2001).

Hierarchical relationships among mice have been studied for decades. Repeated social defeats result in a depressive-like syndrome, including reduced social interactions, decreased body weight, and anxiety-like behavior (Golden et al., 2011; Takahashi et al., 2017). The medial prefrontal cortex (mPFC) plays an important role in social ranking and is subjected to the effects of stress resulting from social defeats (Chaudhury et al., 2013; Covington et al., 2010; Lehmann et al., 2017; Wang et al., 2011). Notably, defeated mice can be further divided into susceptible and resilient (or unsusceptible) subpopulations with behavioral and physiological differences (Krishnan et al., 2007). A decrease in activity-dependent brain-derived neurotrophic factor (BDNF) release in the ventral tegmental area (VTA) promotes resilience (Krishnan et al., 2007). In addition to the mPFC and VTA, the dorsal periaqueductal gray is also involved in social stress-induced behavioral changes (Franklin et al., 2017).

Serotonin or 5-hydroxytryptamine (5-HT) is a monoamine neurotransmitter that is associated with the social state in vertebrate and invertebrate species, but with opposite effects (Edwards and Kravitz, 1997). In monkeys, a low serotonin level is associated with enhanced aggression and a lower social status (Shively et al., 1995). Among crustaceans, American lobsters show a reduced intensity of aggression for at least 1 day after a defeat (Rutishauser et al., 2004). Notably, pharmacologically enhanced 5-HT levels reverse the subordinate state (Huber et al., 1997). Additionally, chronic social defeats in rats downregulate the 5-HT_1A_ receptor in the prefrontal cortex (Kieran et al., 2010).

Studying fruit flies has significantly contributed to our understanding of the neural mechanisms of aggression in recent years. As first clearly described by Jacobs in 1960 (Jacobs, 1960), male fruit flies display stereotypical aggressive behavior (Certel and Kravitz, 2012). Multiple neurotransmitters and neuropeptides, including octopamine, dopamine, 5-HT, acetylcholine, neuropeptide F, and tachykinin (Tk), are involved in aggression in *Drosophila* (Alekseyenko et al., 2013; Asahina, 2017; Asahina et al., 2014; Dierick and Greenspan, 2007; Kim et al., 2017; Zhou et al., 2008). Feeding 5-HT precursors to naive flies promotes their aggression (Dierick and Greenspan, 2007). Furthermore, activating the entire population of 5-HT neurons or a pair of serotonergic posterior lateral protocerebrum neurons (5HT-PLP) increases aggression of naive flies (Alekseyenko et al., 2014).

The aggression in *Drosophila* is also modulated by experience. Loser-loser pairs rarely exhibit aggression even after a period of separation, nor do they form a new stable hierarchical relationship (Yurkovic et al., 2006). The neurons underlying the loser effect are still unknown. Additionally, whether the 5-HT system plays a role in regulating the dynamics of the social state during this process remains unclear. Furthermore, from a behavioral perspective, the loser effect reflects decreased aggression resulting from acts of elevated aggression. Investigation of the connection between the loser effect and aggression at the circuitry level may yield interesting results.

## Results

### Reversal of the Loser Effect by Optogenetics

**Figure 1:**
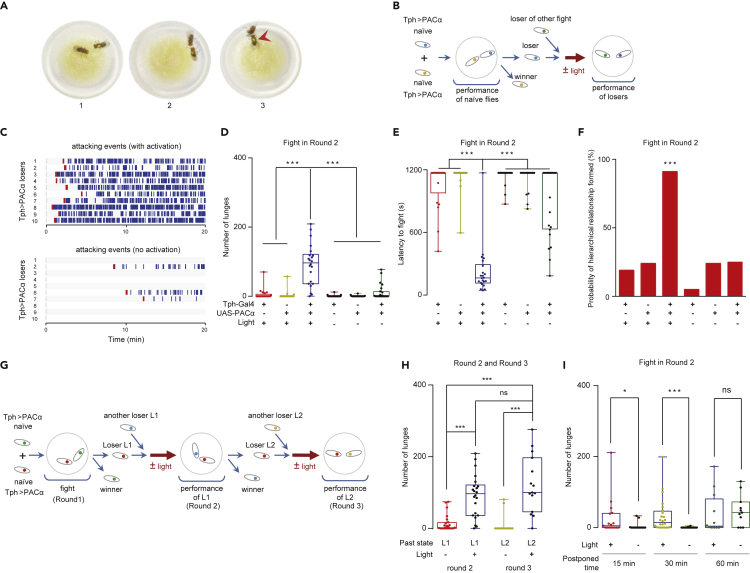
Activation of 5-HT Neurons Promotes Losers to Re-engage in a Fight (A) Two males in the circular fighting chamber fought and formed a winner-loser relationship. Three images show the typical sequence of a fight: (A1) approaching each other, (A2) fighting, and (A3) chasing of the loser by the winner (red arrowhead). (B) Schema of the experimental procedure used to quantify the loser effect. Two singly raised males marked with different colors were pitted in a fighting chamber for Round 1. A loser was then pitted with another loser for Round 2. For motivational treatment, losers received optogenetic stimulation prior to the fight. (C) Raster plots illustrating bouts of lunges in 10 Tph > PACα losers after 80s of photostimulation (top). Control losers did not receive photostimulation (bottom). (D) Optogenetic activation of 5-HT neurons elevated the attack intensity of the losers (n = 21–25). (E) Optogenetic activation of 5-HT neurons reduced the time to fight in loser pairs (n = 21–25). (F) More loser-loser pairs formed hierarchical relationships when their 5-HT neurons were activated (n = 21–25). (G) Schema of the experimental procedure of repeated activation of 5-HT neurons. (H) Photoactivation of 5-HT neurons restored aggression in Tph > PACα losers in Rounds 2 and 3 (n = 24, 22, 14, and 15). (I) Photoactivation of 5-HT neurons triggered a persistent internal state of aroused aggressiveness in Tph > PACα losers (n = 17, 17, 24, 22, 12, and 11). All genotypes and experimental conditions are indicated with the plots. In the box-and-whisker plots (D, E, H, I), the whiskers mark the minimum and maximum, the box includes the 25th to 75th percentiles, and the line within the box indicates the median of the dataset. The Kruskal-Wallis test was performed for (D), (E), (H), and (I), and the chi-square test was performed for (F) (two-tailed χ^2^ = 65.77, df = 5). ns, not significant (P > 0.05); ∗P < 0.05; ∗∗∗P < 0.001. See also Figure S1.

To identify neurons that regulate fighting motivation, we conducted a “fight-club” screen. In this screen, various populations of neurons in socially isolated flies were selectively treated by optogenetics with photoactivated adenylyl cyclase α (PACα), which rapidly increases cyclic adenosine monophosphate levels in the neurons after light stimulation (Schroder-Lang et al., 2007). The fighting behaviors of the males were then observed to select the strains with high fighting intensities. Subsequently, to study the loser effect, we evaluated the effects of activating the candidate neurons with a two-round fighting scheme. When two naive males were pitted in a fighting chamber for Round 1, most naïve-naïve pairs formed a clear winner-loser relationship within 30 min: the loser fled while the winner chased and attacked it (Figure 1A and Video S1). The loser was then paired with a new loser in a new fighting chamber for Round 2 (Figure 1B, for details see Transparent Methods). We quantified the agonistic interactions by the number of lunges (the combined number of lunges by both flies) and the latency to fight (the elapsed time until the first lunge was performed). Among wild-type flies, loser-loser pairs exhibited significantly fewer attacks than did naïve-naïve pairs and fewer loser-loser pairs formed a new winner-loser hierarchy (Figures S1A and S1B).

Supplementary Video S1. After a Fight, a Pair of Flies Formed a Winner–Loser Relationship, Related to Figures 1

We identified neurons labeled by tryptophan hydroxylase (Tph)-Gal4 (Figure S1C) (Park et al., 2006) from the fight-club screen and then investigated the behaviors of losers after photoactivating these neurons. In Round 2, two Tph > PACα losers were introduced into a fighting chamber and immediately received continuous blue light illumination (Figures 1B and S1D). We used an “activation-first and observation-later” approach. After a brief optogenetic treatment (80s), we recorded and scored the aggressive behaviors of the pair for 20 min. The loser-loser pairs exhibited increased attack intensity (Figures 1C and 1D) and decreased aggression latency (Figure 1E). Exposure to light pulses for 80s also produced similar effects on the losers (Figures S1E and S1F). Further cross-validation of our approach with another optogenetic agent, CsChrimson (Klapoetke et al., 2014), also confirmed that activating losers promoted aggression (Figures S1G and S1H). In addition to the elevated fighting intensity, more loser-loser pairs than controls established new hierarchical relationships after photoactivation (Figure 1F).

Interestingly, the new winner in Round 2 could not be readily predicted by the fighting details in Round 1, such as the numbers of attacks on and by the opponent (the winner) or both (Figures S1I–S1K), suggesting that stimulating these neurons effectively decouples aggression or fighting decisions from previous fighting experience. Controllable reversion of loser effects has not been reported in *Drosophila*; therefore, we became interested in exploring the underlying neural mechanisms and relevant molecular signals.

### Activation of 5-HT Neurons Restores Fights in Losers

Tryptophan hydroxylase (Trh or Tph, CG9122) is the rate-limiting enzyme for biosynthesis of 5-HT (Alekseyenko et al., 2010; Coleman and Neckameyer, 2005); we herein refer these Tph-Gal4-labeled neurons as 5-HT neurons. We wondered whether such restoration of aggression in losers by stimulation of 5-HT neurons has any temporal effects. After the fight involving the photoactivated Tph > PACα losers in Round 2, the new losers were collected and paired for Round 3 (Figure 1G). These losers again exhibited a higher fighting intensity when exposed to blue light than did flies without light treatment (Figure 1H). This finding suggests that activation of 5-HT neurons readily induced the losers to fight despite repeated defeats. Interestingly, when Tph > PACα losers were individually stimulated by light for 80 s and kept separated for a time interval before the tests, their motivation to fight lasted for at least 30 min after stimulation, indicating that optogenetic activation of 5-HT neurons changes the internal states of losers (Figure 1I).

**Figure 2:**
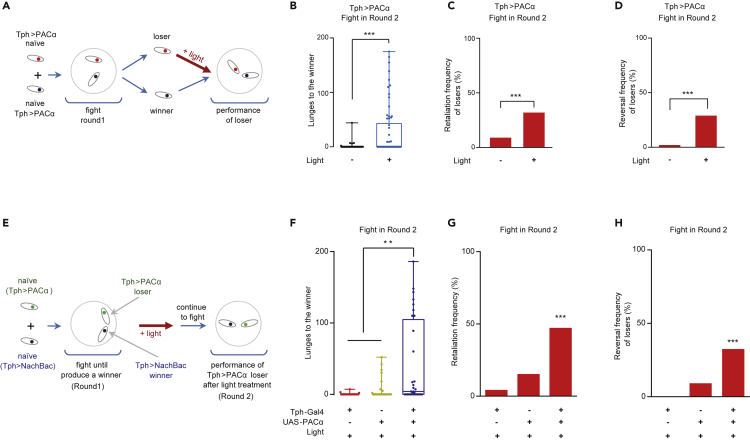
Activation of 5-HT Neurons Promotes Fighting of Losers Against Winners (A) Schema of optogenetic activation of a Tph > PACα loser before pitting it against the familiar winner. (B) The Tph > PACα losers treated with light showed a higher attack intensity (n = 57 and 66). (C) Light treatment of the Tph > PACα losers induced more losers to fight against the familiar winners than those without light treatment (n = 57 and 66). (D) In total, 29% of the losers with activated 5-HT neurons eventually reversed the winner-loser relationship of Round 1 (n = 57 and 66). (E) Schema of the experimental procedure to stimulate losers *in situ*. (F) Optogenetic stimulation increased the attack intensity of Tph > PACα losers to their winners *in situ* (n = 28–34). (G) Optogenetic stimulation increased the retaliation frequency of Tph > PACα losers to their winners (n = 28–34). (H) Optogenetic stimulation increased the reversal frequency of Tph > PACα losers against their winners (n = 28–34). The Mann-Whitney test was performed for (B), Fisher's exact test was performed for (C) and (D), the Kruskal-Wallis test was performed for (F), and the chi-square test was performed for (G) (two-tailed χ2 = 58.16, df = 2) and (H) (two-tailed χ2 = 44.02, df = 2). ∗∗P < 0.01; ∗∗∗P < 0.001. See also Figures S1–S4.

We were interested in determining how a loser with activated 5-HT neurons behaves in Round 2 when facing the same opponent from Round 1 (the familiar winner) (Figure 2A). With light stimulation, these losers showed increased motivation to fight as reflected by a higher attack frequency (Figure 2B), and a higher percentage of losers fought against the familiar winners (Figure 2C). The overall fights involving the photoactivated losers were more intense, suggesting that the fight-backs from losers significantly contributed to the combating dynamic (Figures S1L and S1M). Interestingly, about 30% of the losers with activated 5-HT neurons eventually won Round 2 (Figure 2D) despite their previous loss to the same opponent in Round 1. The reversion of the winner-loser relationship strongly suggests that when comparing physical power or the damage induced by previous fights, neurological factors play pivotal roles in determining the outcome of a fight in flies.

To observe the reversal dynamic, we paired a Tph > PACα male with a Tph > NaChBac male. NaChBac is a voltage-sensitive sodium channel that causes hyperactivation of targeted neurons when overexpressed (Zhou et al., 2008). In Round 1, as soon as the outcome of combat became clear (i.e., when the submissive Tph > PACα fly exhibited continuous retreats), and before the winner-to-be generated more distressing actions on the loser-to-be, we applied blue light directly onto the pair *in situ* instead of removing them from the fighting chamber (Figure 2E). The fights were thereafter allowed to continue, and the actions of familiar winner-loser pairs were analyzed as Round 2. The optogenetic stimulation promptly boosted several aspects of aggression in the Tph > PACα losers against their Tph > NaChBac opponents, which had been winning before the treatment. Besides elevating their attacks (lunging toward the winner, Figure 2F), more losers fought back (increased retaliation frequency, Figure 2G) and ultimately reversed the situation and won the fight (increased reversal frequency, Figure 2H).

### 5-HT Neurons Play Broad Roles in Aggression

We were interested to learn the roles of 5-HT neurons in different aggression processes. Aggression in fruit flies was strongly affected by rearing conditions. In Round 1, males raised in isolation (singly raised or socially isolated) promptly fought against each other (Figure S1A), whereas males raised in groups (group-raised) hardly fought when pitted in a fighting chamber (Figure S2A). Notably, photoactivating 5-HT neurons promoted intense fights in a pair of group-raised males (Figure S2B). In addition, socially isolated males exhibited increased aggression when 5-HT neurons were activated (Figures S2C and S2D). The inducing effects of light-activated 5-HT neurons on aggression in naive flies are consistent with previous reports using thermogenetics (Alekseyenko et al., 2010, 2014).

We next investigated whether activation of 5-HT neurons induces aggression arousal, which overcomes the requirement for environmental factors or conspecific cues to mount a fight, as was shown in Tk-Gal4 neurons (Asahina et al., 2014). Nutrient-rich food in the fighting chamber was ordinarily necessary for males to engage in a fight. However, activation of 5-HT neurons promoted fights in a chamber without food (Figure S2E). Strikingly, flies with activated 5-HT neurons still fought in the dark despite the fact that intact vision has been considered essential for aggression in *Drosophila* (Figure S2F).

The loser flies in Figure 1B resulted from a fight between singly raised flies. Activation of 5-HT neurons in group-raised flies promoted aggression and generated losers that enabled us to test losers previously raised in groups (Figure S2B). As shown in Figures S2G–S2I, photoactivation of 5-HT neurons in Round 2 restored aggression in the group-raised Tph > PACα losers. Furthermore, photoactivation of 5-HT neurons in the Tph > PACα winners from Round 1 elevated their aggression as well (Figures S2J and S2K). Together, 5-HT neurons participate in the control of multiple forms of aggression.

### Losers Regain Motivation to Fight

The main indicator of reversal of the loser effect is elevated aggression in the losers (Figure 2). The broad influence of 5-HT neurons on multiple forms of aggression prompted us to address whether an elevation in aggression would generally overcome the loser effect. For this purpose, we tested the outcomes of activating 5HT-PLP neurons (Alekseyenko et al., 2014) and Tk-Gal4 neurons (Asahina et al., 2014), both of which reportedly promote aggression when activated in singly raised or group-raised flies. In Round 1, activating 5HT-PLP neurons weakly promoted aggression in socially isolated males, whereas activating Tk-Gal4 neurons strongly elevated aggression in these flies (Figures S3A, S3B, S3E, and S3F). Notably, in Round 2, activation of these aggression-promoting neurons in losers did not increase their tendency to escalate conflicts, regardless of their weak or strong effects in Round 1 (Figures S3C, S3D, S3G and S3H). Therefore, indiscriminately elevating aggression is not sufficient to motivate losers to fight in future agonistic encounters unless the responsible neurons are specifically targeted.

Fighting in flies exhibits a strong “initiator effect,” in which the fly that instigates the first attack has a higher probability of eventually winning the combat (Chen et al., 2002). Because the first attack is usually the first actual physical contact between the pair, this behavioral feature represents a high motivation for aggression. We analyzed the outcomes of Round 2 in 53 familiar loser-winner pairs from Round 1 (Figure S4). After having received optogenetic treatment prior to Round 2, 26% (14 of 53) of Tph > PACα losers from Round 1 (L1) won Round 2; in comparison, only 4% of untreated L1 (controls) won Round 2. Notably, 36% (19 of 53) of L1 with photoactivated 5-HT neurons initiated the fight in Round 2, whereas only 4% (1 of 27) of non-activated losers did so. Among these Tph > PACα losers that initiated the fight in Round 2, 74% (14 of 19) won the fight, in comparison to 97% (33 of 34) of the familiar winners and 100% (26 of 26) of winners in the control group that initiated and won the fights in Round 2. Therefore, in contrast to the losers with activated Tk-Gal4 neurons, which were unwilling to participate in a fight at all, the losers with activated 5-HT neurons would start a fight even when they were incapable of winning it. The chance of victory for these losers is possibly determined by both mental and physical factors.

### Serotonin Projection Neurons and P1 Neurons Elicit Losers to Fight

**Figure 3:**
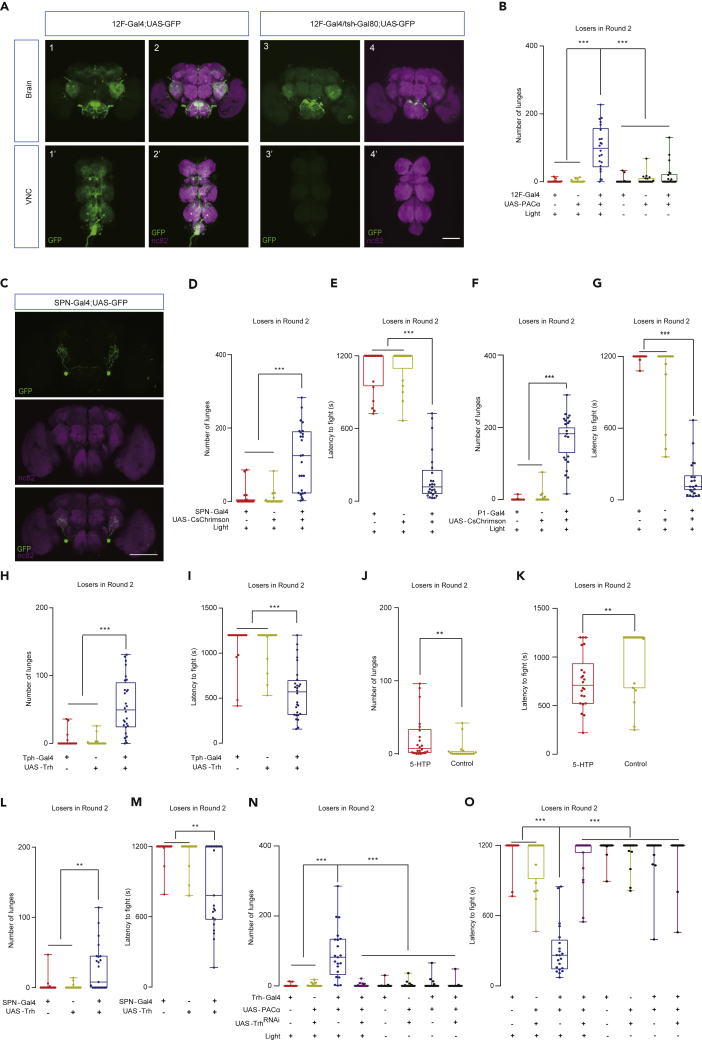
Serotonergic Signal is Functionally Sufficient for Overcoming the Loser Effect (A) Expression patterns of 12F-Gal4/UAS-mCD8:GFP (A1-A2′) and expression patterns of 12F-Gal4 driving UAS-GFP in the presence of Tsh-Gal80 (A3-A4′). (B) Photoactivation of 12F-Gal4-labeled 5-HT neurons promoted attacks in losers (n = 22–24). (C) Expression pattern of SPN-split-Gal4/UAS-mCD8:GFP. (D and E) Optogenetic activation of SPNs induced aggression (D) and rendered short latency to fight (E) in losers (n = 20–25). (F and G) Activation of P1 neurons promoted aggression with increased attack intensity (F) and reduced latency to fight (G) in losers (n = 21–22). (H and I) Elevated 5-HT levels in 5-HT neurons induced aggressive actions (H) and reduce the latency to fight (I) in loser pairs (n = 21–28). (J and K) Pharmacologically increasing the 5-HT levels promoted aggression (J) and decreased the latency to fight (K) in losers (n = 19–22). (L and M) Elevating the serotonin level in SPNs promoted aggression (L) and decreased the latency to fight (M) in losers (n = 21–24). (N and O) Simultaneously activating Tph-GAL4 neurons and reducing the serotonin level in the same neurons blocked the loser from gaining aggression (N) and shortening the latency to fight (O) (n = 21–23). The Kruskal-Wallis test was performed for (B), (D)-(I), and (L)-(O); the Mann-Whitney test was performed for (J) and (K). ∗∗P < 0.01; ∗∗∗P < 0.001. Scale bar, 100 μm. See also Figures S5–S6.

We next characterized the 5-HT neurons responsible for overcoming the loser mentality. Tph > GFP signals in an adult male revealed more than 100 cells in the brain and ventral nerve cord (VNC). We evaluated additional 5-HT Gal4 lines, including 12F-Gal4, which labeled subpopulations of 5-HT neurons (Alekseyenko et al., 2010) (Figure 3A). Optogenetic activation of 12F-Gal4 neurons restored the aggression of the losers (Figures 3B and S5A). We then combined this Gal4 with various Gal80s (Zhan et al., 2016) to further reduce the responsible neurons. As shown in Figures 3A, S5B, and S5C, the cholinergic-positive serotonergic neurons in the brain were functionally sufficient for overcoming the loser effect.

We next used split Gal4 lines and identified a pair of serotonin projection neurons (SPNs) (Scheunemann et al., 2018), which were located in the gnathal ganglia and projected to the central brain (Figure 3C). Optogenetic activation of these SPNs increased aggression in socially isolated males (Figures S5D and S5E), whereas silencing these neurons by tetanus toxin light chain (TNT) (Sweeney et al., 1995) did not affect their fight intensity (Figures S5F and S5G). These results indicate that SPNs are functionally sufficient but not necessary for aggression in Round 1. Notably, activating SPNs in losers induced intense fights via CsChrimson (Figures 3D and 3E) or PACα (Figures S5H and S5I), demonstrating that activating a pair of 5-HT neurons (not a pair of 5HT-PLP neurons) is sufficient to overcome the loser effect.

Besides 5-HT neurons, we continued to identify other neurons that mediate reversal of the loser effect. We began with neurons known to promote aggression in Round 1, including Tk, 5HT-PLP, and P1 neurons (Hoopfer et al., 2015). Interestingly, in Round 2, the activation of P1 neurons in losers increased their attack frequency (Figure 3F) and shortened the latency to fight (Figure 3G). The differential effects of Tk, 5HT-PLP, SPNs, and P1 neurons suggest that aggression-promoting neurons function differently in promoting losers to fight.

### 5-HT1B as a Downstream Receptor to Overcome the Loser Effect

We further investigated whether 5-HT is involved in the loser effect. As shown in Figures 3H, 3I, and S5J, elevated 5-HT levels via overexpression of Tph in Tph-Gal4-labeled neurons motivated the losers to fight. In addition, we fed flies a 5-HT precursor, 5-hydroxytryptophan (5-HTP) (Dierick and Greenspan, 2007), to pharmacologically increase the 5-HT levels. Treatment with 50 mM 5-HTP was sufficient to restore the fighting of losers (Figures 3J and 3K). Additionally, increasing the 5-HT levels in SPNs alone motivated losers to fight again (Figures 3L and 3M). Similarly, 5-HTP treatment promoted aggression in group-raised males in Round 1 (Figures S6A–S6D), suggesting a role of 5-HT in aggression in Round 1. This is also confirmed by genetic evidence. Overexpressing Trh in either Tph neurons or SPNs increased the level of aggression in singly raised males in Round 1 (Figures S6E–S6H).

To confirm that 5-HT is necessary to mediate reversal of the loser effect, we activated 5-HT neurons in losers with decreased 5-HT levels. In the presence of Trh^RNAi^ (Albin et al., 2015), photoactivation of 5-HT neurons failed to restore the loser effect (Figures 3N and 3O), suggesting that while 5-HT neurons can use multiple neurotransmitters; 5-HT is responsible for overcoming the loser effect. These results suggest that 5-HT in the 5-HT neurons is necessary and sufficient for reversal of the loser effect, prompting further investigation of downstream receptor and receptor neurons.

**Figure 4:**
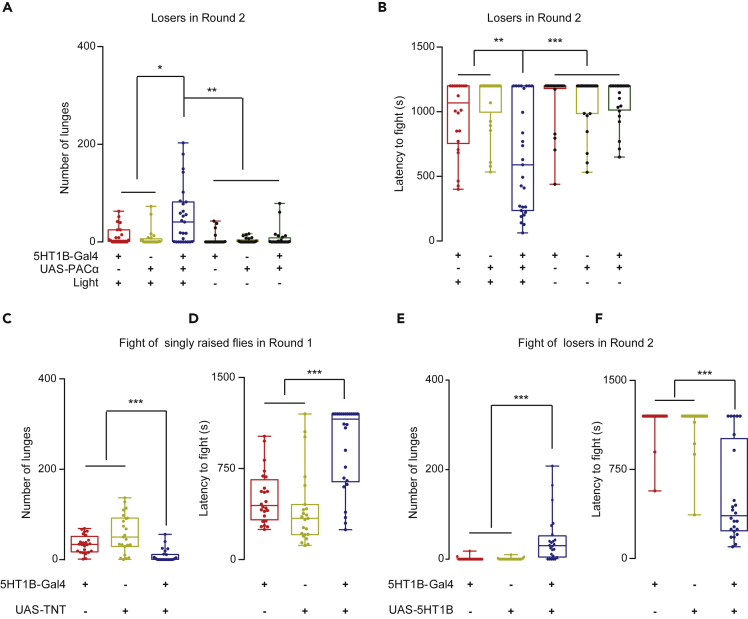
5-HT1B Neurons and 5-HT1B Receptors are Responsible for Restoring Aggression in Losers (A and B) Optogenetic activation of 5-HT1B neurons promoted aggression (A) and decreased the latency to fight (B) in losers (n = 22–27). (C and D) Inhibition of synaptic transmission of 5-HT1B neurons decreased the attack intensity (C) and increased the latency to fight (D) in socially isolated males (n = 23–25). (E and F) Overexpressing 5-HT1B receptor in 5-HT1B neurons induced aggression (E) and decreased the latency to fight (F) in losers (n = 22–24). The Kruskal-Wallis test was performed for (A)-(B) and (E)-(F), and one-way ANOVA was performed for (C) and (D). ∗P < 0.05; ∗∗P < 0.01; ∗∗∗P < 0.001. See also Figure S7.

Five 5-HT receptors have been identified in *Drosophila*: 5-HT1A, 5-HT1B, 5-HT2A, 5-HT2B, and 5-HT7 (Gasque et al., 2013). 5-HT1A neurons have been shown to participate in aggression by acting downstream of 5HT-PLP neurons (Alekseyenko et al., 2014); however, the functions of other receptor neurons in aggression are unknown. Notably, optogenetic activation of 5-HT1B neurons (Yuan et al., 2005) in losers was sufficient to restore their motivation to fight (Figures 4A, 4B, S7A, and S7B), suggesting that 5-HT1B neurons are also involved in overcoming the loser effect. We could not test the functions of 5-HT2A and 5-HT2B because the progenies of both for several optogenetic manipulations were not viable.

Activation of 5-HT1B neurons increased aggression in group-raised males (Figure S7C), while silencing these neurons by TNT suppressed aggression in singly raised males (Figures 4C and 4D). These findings indicate that 5-HT1B neurons are functionally sufficient and necessary for aggression. Notably, the strength to elevate aggression in naive flies by activated 5-HT1B neurons was relatively low, as determined by the attack intensity (Figure S7C). However, the same treatment was still sufficient to motivate losers to fight in Round 2 (Figure 4B), suggesting that in general, high aggression in naive flies does not simply correlate with restoring an adequate motivation to fight in losers.

To evaluate the role of the 5-HT1B receptor in overcoming the loser effect, we overexpressed the 5-HT1B receptor in 5-HT1B neurons. Similar to activating the 5-HT1B neurons, increasing the levels of the 5-HT1B receptor both elevated aggression in naive males (Round 1, Figures S7D and S7E) and restored the aggression of losers (Round 2, Figures 4E, 4F, and S7F). These data suggest that at the molecular level, both 5-HT and its receptor 5-HT1B are responsible for overcoming the loser effect.

### R2/R4m Neurons Are Downstream Targets of 5-HT Neurons

**Figure 5:**
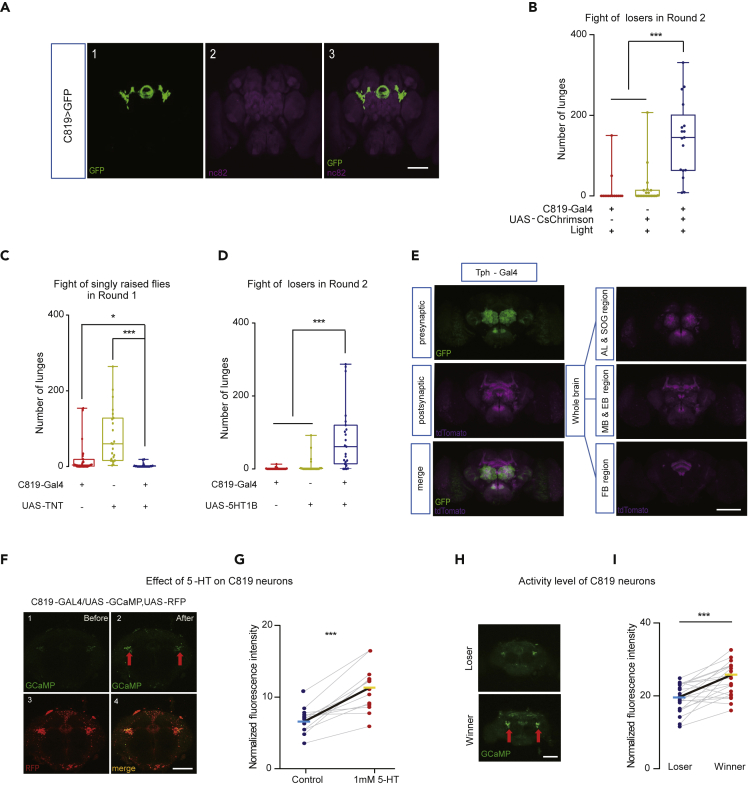
R2/R4m Neurons in the Ellipsoid Body Are Responsible for Overcoming the Loser Effect (A) Expression patterns of C819-Gal4/UAS-mCD8:GFP revealed strong signals in the R2/R4m neurons of the EB region. (B) Activation of C819-Gal4-labeled R2/4m neurons promoted the attack intensity of the losers (n = 14–22). (C) Inhibition of synaptic transmission of C819 neurons decreased the attack intensity of socially isolated males (n = 23–25). (D) Enhanced 5-HT1B receptor level in R2/R4m neurons restored aggression in losers (n = 22–24). (E) 5-HT neurons were visualized by Tph > myrGFP signals (green). The *trans*-Tango approach revealed the postsynaptic neurons in AL, SOG, MB, EB, and FB (mtdTomato signal, purple). (F) The direct application of serotonin hydrochloride increased the calcium level in C819 neurons. The confocal images showed the GCaMP signals in the R2/R4m neurons of the EB region before 5-HT delivery (F1) and after 5-HT delivery (F2). Panel F3 shows the corresponding cell clusters in this region, which were identified according to the RFP signal (red). Panel F4 shows the overlay staining patterns of F2 and F3. (G) Quantification of mean GCaMP signals (normalized to RFP signals) in C819 neurons before and after 5-HT application (n = 13). (H and I) Losers displayed lower calcium levels in C819 neurons than the corresponding winners (n = 20–21). The Kruskal-Wallis test was performed for (B)-(D), and the paired t test was performed for (G) and (I). ∗P < 0.05; ∗∗∗P < 0.001. Scale bar, 100 μm. See also Figure S8.

We next asked which subgroup of 5-HT1B neurons is involved in the loser effect. Different populations of 5-HT1B neurons were located in the mushroom body (MB), ellipsoid body (EB), and VNC (Figures S8A1–S8A4). With the use of Gad-Gal80 (Yuan et al., 2014) and MB-Gal80 to prevent the 5-HT1B neurons in the VNC and MB, respectively, from being manipulated (Figures S8A5–S8A12), our results indicated that optogenetic activation of the 5-HT1B neurons in the EB alone is sufficient for overcoming the loser effect (Figures S8B–S8E). To investigate the subgroup of EB neurons responsible for this effect, we conducted a small-scale screen and identified C819-Gal4 (Guo et al., 2014). C819-Gal4-labeled neurons were located in the R2/R4m region of the EB (Figures 5A and S8F). Activation of C819 neurons promoted fighting of singly raised males (Figure S8G) and losers (Figures 5B and S8H), whereas silencing of C819 neurons by TNT decreased the motivation to fight in singly raised males (Figures 5C and S8I). Furthermore, overexpressing the 5-HT1B receptor in C819 neurons promoted fighting of singly raised males (Figure S8J) and restored the aggression of losers (Figures 5D, S8K and S8L), suggesting that C819 neurons use 5-HT1B receptors to mediate the signaling for overcoming the loser effect and for elevating aggression.

To visualize the structural correlation between the C819 neurons and the 5-HT neurons, we utilized the *trans*-Tango system, a tool for anterograde trans-synaptic circuit tracing (Talay et al., 2017). As shown in Figure 5E, in flies bearing the *trans*-Tango components, Tph-Gal4 drove myrGFP expression (green) in presynaptic neurons (5-HT neurons) and induced mtdTomato expression (red) in postsynaptic neurons, which were located in the EB and other regions. The *trans*-Tango signals in the EB area included the arborization pattern of C819 neurons, suggesting that these EB neurons are likely a target of 5-HT neurons.

The 5-HT1B receptor in *Drosophila* exhibits the most substantial homology (approximately 41%) to the human 5-HT_1A_ receptor (Tierney, 2001). Previous researchers have proposed that mammalian 5-HT1A is GI alpha subunit (Giα)-coupled; thus, serotonergic signaling through this kind of receptor would suppress the receptor neurons (Nichols and Nichols, 2008). However, the assumption that the signaling of the *Drosophila* 5-HT1B receptor is similar to that of the human 5-HT1A receptor conflicts with two behavioral results. First, activating 5-HT neurons (Figures 1D and 1E) and activating 5-HT1B neurons (Figures 4A and 4B) had similar effects on reversing the loser effect. Second, both increasing the level of 5-HT (Figures 3H and 3I) and increasing the level of the 5-HT1B receptor (Figures 4E and 4F) overcame the loser effect. To further explore 5-HT signaling, we directly measured the activity of 5-HT1B neurons upon stimulation of 5-HT. As shown in Figures 5F, 5G, and S8M, when applied to a brain explant, 1 mM of 5-HT hydrochloride (Haynes et al., 2015) effectively increased the activity of the C819 neurons. Therefore, both behavioral and imaging data suggested that release of 5-HT causes activation of the 5-HT1B neurons in the fly brain.

The loser effects in *Drosophila* last for three hours (after a single defeat) to one day (after repeated defeats) (Trannoy et al., 2016); such a long timescale could be due to physiological, endocrinal, or neurological changes. As a first step to identify the traces left by the fighting experience, we examined the changes in the R2/R4m neuron activity before and after the fights with the calcium imaging method. Following a fight, the C819 neurons in losers exhibited lower calcium signals (GCaMP fluorescence) in losers than in winners (Figures 5H and 5I), suggesting that social defeat (or victory) is closely associated with the relative activities of these neurons in losers (or winners). Notably, the activity of the C819 neurons in either winners or losers was higher than that in controls (socially isolated naive flies) (Figure S8N). Engaging in a fight might also increase the basal activity of these neurons, which are responsible for promoting aggression in naive flies. Although the constant movements and actions of both flies made it challenging to monitor the dynamic changes in neuronal activities throughout a fight, the above measurements at the endpoints demonstrated that the C819 neurons are modulated by both the process and the output of fighting.

**Figure 6:**
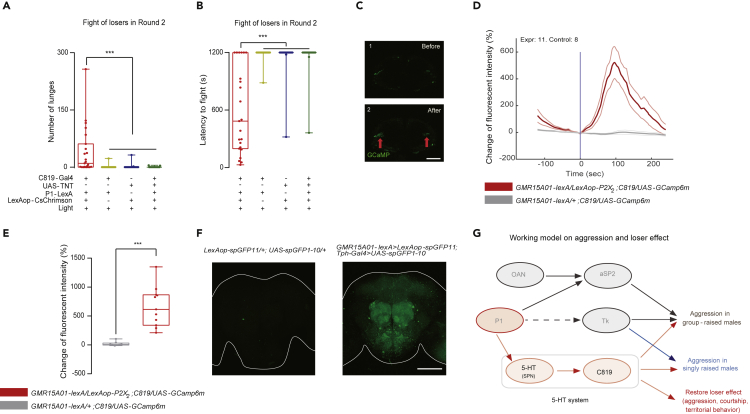
P1 Neurons Act Upstream of 5-HT Signaling (A and B) Activating P1 neurons (labeled by R15A01-LexA) while simultaneously silencing C819 neurons did not induce aggression in losers, reflected by the low fighting intensity (A) and long latency to fight (B) (n = 23). (C–E) Visualization of functional connectivity between P1 and C819 neurons by applying ATP directly to the brain. P1 neurons responded to ATP and resulted in increased GCaMP signals in C819 neurons. Genotypes are indicated at the bottom. (C) Images of confocal sections of GCaMP signals in the C819 neurons: before ATP delivery (C1) and after ATP delivery (C2). (D) Traces of fluorescent intensities of C819 neurons before and after P1 activation. Time zero is indicated by a blue line. Red lines label the signals from the experimental group, and gray lines label the control group. Thick lines represent the average signals, and thin lines indicate one standard deviation (n = 8–11). (E) Maximal changes of fluorescent intensity in C819 neurons after P1 activation (n = 8–11). (F) GRASP signals revealed structural connections between P1 neurons and 5-HT neurons. Left: an image of the brain of control flies with spGFPs only. Right: R15A01-LexA drove the presynaptic component (spGFP11), and Tph-Gal4 drove the postsynaptic component (spGFP1–10). (G) Working model of the 5-HT system on controlling aggression and loser mentality at the circuit level. Besides arousing or increasing aggression across flies with different social experiences, the 5-HT system also regulates the aggression restore in losers, with P1 neurons acting upstream. The Kruskal-Wallis test was performed for (A) and (B); the t test was performed for (E). ∗∗∗P < 0.001. Scale bar, 100 μm.

In an effort to map the neural circuit of motivating losers, we studied the hierarchical order of the 5-HT system and P1 neurons. Considering that inactivating or ablating P1 neurons had no impact on the aggression of singly raised males (Hoopfer et al., 2015) whereas inactivating 5-HT receptor neurons effectively blocked aggression (Figures 4C and 5C), it is likely that the 5-HT system acts downstream or in parallel with P1 neurons in aggression control in losers. Therefore, we quantified the synergistic effects of simultaneously activating P1 neurons and inactivating the 5-HT system. As shown in Figures 6A and 6B, losers with both activated P1 neurons (by optogenetics) and silenced C819 neurons (by TNT) completely failed to recover their aggression. The fact that silencing the C819 neurons abolished the loser-reversal effect evoked by activating P1 neurons suggests that P1 neurons are functionally upstream of the 5-HT system.

To visualize the functional connectivity, we transiently activated P1 neurons with ATP through an ATP-dependent depolarizing ion channel, P2X_2_ (Chen et al., 2017; Lima and Miesenbock, 2005; Liu et al., 2019b), while simultaneously recording calcium signals from the C819 neurons labeled with GCaMP6. We found that 2.5 mM ATP induced robust calcium responses in the cell bodies of C819 neurons (Figures 6C–6E), whereas no signals were induced in the controls lacking P2X_2_ in P1 neurons (Figures 6D and 6E). This finding suggests that active P1 neurons can lead to activation of C819 neurons. We next checked for direct connections between P1 neurons and 5-HT neurons using the GFP Reconstitution Across Synaptic Partners (GRASP) method (Feinberg et al., 2008). GFP puncta were observed in multiple brain regions, and the most prominent signals were located in the antenna lobes and the subesophageal zone (Figure 6F). No GFP signals were detected in the brains of the control group (Figure 6F). Together, these results suggest a pathway from P1 neurons to 5-HT1B neurons for mediating reversal of the loser effect (Figure 6G).

### Upregulating 5-HT Signaling Broadly Rescues Suppressive States of Losers

**Figure 7:**
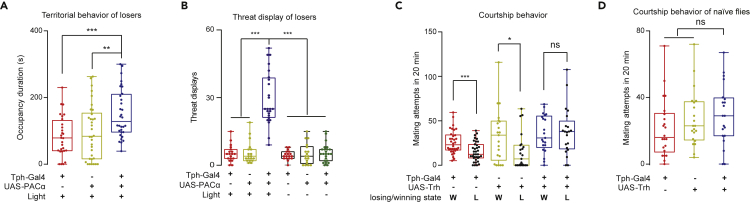
Suppressive Loser Mentality is Effectively Removed by Increasing Serotonin Signaling (A) Optogenetic activation of Tph-Gal4 neurons in losers rendered a longer occupancy duration for food patches as an indication of territorial behavior (n = 28–34). (B) Activating Tph-Gal4 neurons increased threat displays of losers (n = 19–24). (C and D) The losers displayed lower courtship motivation than the winners (C). Elevating serotonin levels restored courtship motivation of losers (C) (n = 21–28) but did not affect the courtship motivation of singly raised males (D) (n = 20–22). One-way ANOVA was performed for (A)-(D). ns, not significant (P > 0.05); ∗P < 0.05; ∗∗P < 0.01; ∗∗∗P < 0.001.

The outcomes of conflicts induce behavioral modifications in many animals. Therefore, we characterized the behavioral changes of the loser flies and tested whether activation of 5-HT neurons could overcome these depressive effects as well. First, losing a fight resulted in reduced territorial behaviors (Zhou et al., 2008). We paired a Tph > PACα with a Tph > NaChBac to produce a Tph > PACα loser (Figure 2E), which exhibited a decreased duration of occupation of a food patch. Optogenetically stimulating the Tph > PACα loser *in situ* rapidly restored its display of territorial behaviors (Figure 7A).

Second, in conflict situations, threat displays are often elicited to signal an intent to escalate the conflict. Recent reports have shown that in *Drosophila*, the neurons in the anterior inferior protocerebrum regulate threat displays, and threat displays are modulated by social defeat (Duistermars et al., 2018; Yuan et al., 2014). We found that photoactivation of 5-HT neurons greatly enhanced the level of threat displays (Figure 7B).

Third, a loser male showed fewer courtship behaviors toward a female (Kim et al., 2018; Teseo et al., 2016). With overexpression of Trh in Tph-Gal4-labeled 5-HT neurons, the elevated 5-HT levels re-established the courtship motivation of losers (Figure 7C) while having no effects on naive flies (Figure 7D).

Together, social defeats in *Drosophila* result in modification of behaviors across other contexts, suggesting a persistent and broadly suppressive mental state (“loser mentality”) that can be successfully overcome by increasing serotoninergic signaling.

## Discussion

In this study, we showed that an increase in 5-HT signals effectively overcomes the low motivational state in male flies after losing a fight. Optogenetic activation of 5-HT neurons promoted aggression of singly raised males and, more importantly, restored the motivation to fight in loser males. While P1 neurons acted upstream, 5-HT1B receptor neurons in the EB acted downstream of 5-HT neurons in overcoming the loser effect. Enhanced expression of 5-HT and 5-HT1B motivated the losers to fight. Notably, the suppressive state induced by losing a fight, reflected in the failure to display territorial behavior, decreased threat displays, and decreased mating attempts, was also rescued by elevated serotonergic signals.

### Loser Effect and Its Reversal

Social experiences are known to generate long-lasting effects on behavior. In mice, repeated social defeats lead to chronic social defeat stress with behavioral deficits resembling depression (Covington et al., 2010; Golden et al., 2011; Takahashi et al., 2017). Investigations of these deficits by chronic social defeat stress and their reversion have produced many leads. Calorie restriction reverses the behavioral deficits, and orexin plays an essential role in this process (Lutter et al., 2008a). Moreover, ghrelin and the growth hormone secretagogue receptor can defend animals against stress-induced depression-like symptoms (Lutter et al., 2008b). Social defeat stress also downregulates transcription of BDNF, likely via increased histone methylation (Tsankova et al., 2006), while both hyperacetylation and downregulation of histone deacetylase reverse this process (Tsankova et al., 2006). Interestingly, optogenetic inhibition of VTA dopamine neurons projecting to the nucleus accumbens (NAc) in susceptible mice render them resilient at the time of illumination (Chaudhury et al., 2013). Dominant mice show strong synaptic strength in the mPFC, and elevating the synaptic transmission in the mPFC increases the ranks within the group (Lehmann et al., 2017; Wang et al., 2011). Moreover, unselectively stimulating neurons in the mPFC using optogenetics generates strong and long-lasting antidepressant-like effects, restoring the social interactions in susceptible mice (Covington et al., 2010).

In *Drosophila*, chronic sexual rejection suppresses courtship behavior and increases intake of ethanol in males (Mehren et al., 2004; Shohat-Ophir et al., 2012). Interestingly, after losing a single fight, a male fly exhibits reduced aggression and courtship (Teseo et al., 2016; Yurkovic et al., 2006). These negative social experiences modulate multiple behaviors beyond the context the flies initially witnessed, leading to depressive behavioral states. However, the underlying neural mechanisms are largely unknown.

The loser effect might have adaptive value by preventing escalation, reducing energy expenditure, and lowering the risk of further injury of the losers. Nevertheless, losing a fight not only reduces the chance to compete for resources or mates but also generates pleiotropic depressive effects unrelated to aggression. For example, social defeat decreases territory-holding power in *Drosophila* losers (Yurkovic et al., 2006; Zhou et al., 2008). However, compared with aggression, investigation of the loser effect is rare in *Drosophila* and other animal models. Both the neural circuit responsible for the behavioral changes and the active mechanism of overcoming the loser mentality are unclear.

The loser effect in *Drosophila* lasts for hours after a single defeat (or days after repeated defeats) (Kim et al., 2017; Trannoy et al., 2016; Yurkovic et al., 2006). Losers' low motivation to fight or mate during this period suggests that experiencing or accepting a single social defeat induces prolonged depressive after-effects on subsequent behaviors, some of which are unrelated to aggression or fighting. We designated this persistent depressive state “loser mentality.” More investigations are needed to identify additional behaviors affected in losers and to reveal the common neural substance that likely acts in a top-down manner to modulate distinct behaviors. We identified critical neurons that, once optogenetically activated, can revert the loser's behavioral state. Two optogenetic agents, PACα and CsChrimson, were used, and the results validated each other on key conclusions. Because both agents have rapid and reversible kinetics (<1 s) (Schroder-Lang et al., 2007; Klapoetke et al., 2014), the “activation-first and observation-later” experimental scheme using these activators decouples the behavioral consequence from the cause (brief photoactivation of neurons) along time to help in the probing of persistent effects.

The present study has revealed that enhancing serotoninergic signaling can effectively rescue multiple depressive behaviors after social defeats. A neural pathway from P1 neurons to 5-HT neurons and then to 5-HT1B neurons is associated with motivational changes in losers. Interestingly, the neuronal activity in 5-HT1B neurons was lower in losers than in winners, providing a starting point from which to investigate the neurological modification of the brain due to the loser effect.

### Blindly Elevating Aggression Is Not Sufficient to Motivate Losers

From a behavioral perspective, aggression and the loser effect are closely intertwined, further complicating investigation of these processes. The loser effect is the consequence of losing an aggressive encounter, while the critical indicator of reversal of the loser effect is elevated aggression (in losers). Interestingly, a recent finding in beetles suggested a separation between fighting ability and the loser effect (Okada et al., 2019).

The multiple effects of 5-HT neurons on aggression are intriguing at first glance. In the present study, we showed that 5-HT neurons, tested as the entire population (labeled by Tph-Gal4) or subpopulation (labeled by 12F-Gal4 and SPN-Gal4), and 5-HT1B receptor neurons promote aggression in flies of different social backgrounds (group-raised naive flies, singly raised naive flies, and winners), but also arouse the losers to fight.

Nevertheless, increasing aggression is not identical to reversal of the loser effect. Our behavior analysis showed that indiscriminately activating aggression-promoting neurons, such as Tk-Gal4 neurons, did not overcome the loser effect despite their strong aggression-promoting effect on naive flies. Thus, there is a qualitative difference, rather than just a quantitative difference, between these aggression-promoting neurons. In losers, the aggression-restoring effect goes beyond the typical aggression-promoting effect in naive flies, relying on specific serotonergic signals along the P1/SPN/5HT-1B axis, not Tk-Gal4 or 5HT-PLP neurons. Therefore, as shown in Figure 6G, 5-HT/5-HT1B signaling plays dual roles at the circuit level. This pathway is within the shared section for arousing aggression in naive, winner, and loser flies, while it is also responsible for overcoming the loser's motivations beyond fighting back (such as courtship).

Generally, there are distinct situations that elicit aggressive behaviors in an animal, such as competing for territory or a chance to mate, protecting young offspring, or defying a predator; however, whether different types of aggression exist remains unclear. An apparently similar behavioral expression, such as elevation of aggression, could be due to different signaling pathways. By analyzing the fights in different contexts (Rounds 1 vs. 2), our data suggest that for various ultimate causes, aggressive behaviors with seemingly identical characteristics might arise from different neural states based on different neural circuits (but with extensive overlap).

### 5-HT Signaling in Aggression and Loser Effect

Activating the entire population (Tph) or a pair of serotonergic neurons (5HT-PLP) reportedly plays regulative roles in promoting aggression in naive *Drosophila* (Dierick and Greenspan, 2007). Along this line of investigation, the present study showed that 5-HT signaling broadly promotes aggression in flies of different social experiences and wining-losing statuses. On the receptor side, 5-HT1A neurons suppress aggression in naive flies (Alekseyenko et al., 2014). Our data reveal that downstream of 5-HT neurons, the 5-HT1B neurons in the EB promote aggression in naive flies (singly raised or group-raised), thus extending the repertory of 5-HT receptor neurons in regulating aggression.

More importantly, our results demonstrate that elevating the 5-HT signals motivates loser flies to fight again. In contrast to 5HT-PLP neurons, another pair of 5-HT neurons (SPNs) is sufficient to induce the losers to fight when activated. Interestingly, these losers are not merely responding to aggressive opponents; instead, the losers with activated 5-HT neurons flip the “fight or flight” decision and initiate the first strike before the opponents. Together, our results suggest that the SPN/5-HT1B pathway rather than the 5HT-PLP/5-HT1A pathway plays a decisive role in motivating the losers.

Would the 5-HT signaling revealed here also rescue low motivational states of other causes? With a paradigm inducing a depression-like state in *Drosophila*, Ries et al. showed that serotonin uses different downstream receptors (5-HT1A in the α-/β-lobes and 5-HT1A in the γ-lobe of the MB) for opposite aspects of behavior relief (Ries et al., 2017). Flies display a behavioral state called “learned helplessness” after uncontrollable stressful events, and a similar behavioral state in mammals has been linked to 5-HT (Yang et al., 2013). Serotonin is critical in regulating feeding and sleep in naive flies. It would be interesting to determine whether loser flies also depend on 5-HT signaling to recover from related changes after defeats (Albin et al., 2015; Liu et al., 2019a; Qian et al., 2017; Yuan et al., 2005).

Our behavioral and imaging data suggest that in contrast to the 5-HT1A receptor, the 5-HT1B receptor activates postsynaptic neurons upon receiving the 5-HT signal. The evidence from a study of the action of serotonin on the MB supported this view. In the depression-like model of *Drosophila*, initiating behavioral activity is enhanced by 5-HT1A neurons but inhibited by 5-HT1B neurons despite the fact that both receive input from upstream serotonergic Trh493 neurons (Ries et al., 2017). 5-HT1A and 5-HT1B might utilize distinct intracellular mechanisms for signal transduction. As a GPCR, the 5-HT1B receptor of the EB neurons may couple to other types of G-proteins or intracellular signaling molecules instead of Giα (Nichols and Nichols, 2008).

### Possible Conserved Functions of 5-HT Signaling

As shown in recent studies, 5-HT modulates aggression and motivational states in *Drosophila*. Intriguingly, the downstream signaling of 5-HT quickly splits into opposing pathways. Our results indicate that activating 5-HT1B neurons promotes aggression whereas activating 5-HT1A neurons suppresses aggression (Alekseyenko et al., 2014). Similarly, 5-HT1A and 5-HT1B neurons in the MB receive the same input but generate opposite outputs (Ries et al., 2017). A recent study on the downstream neural circuit of 5HT-PLP neurons revealed two types of 5-HT1A neurons with opposite effects on aggression (Alekseyenko et al., 2019). The bifurcation of 5-HT signals into downstream pathways with opposing behavioral expressions implies that the neural circuits of motivations consist of opposing branches accomplishing a delicate balance. The complexity of 5-HT signaling probably originates from an ancient and simple pathway, the function of which is still unclear.

5-HT1A and 5-HT1B in *Drosophila* are homologs of mammalian 5-HT_1A_ (Tierney, 2001). The 5-HT system is associated with aggression and the social state in vertebrate species. 5-HT and its receptors, the 5-HT1 and 5-HT2 families, are considered vital regulators of aggression in rodents based on pharmacological evidence (Edwards and Kravitz, 1997; Olivier et al., 1995). For example, a study showed that the 5-HT1A receptor in the prefrontal cortex was downregulated by chronic social defeats in rats (Kieran et al., 2010). Intriguingly, while systemic administration of 5-HT_1A_ receptor agonists decreases aggression in rodents, local injection of 5-HT_1A_ and 5-HT_1B_ receptor agonists in either the mPFC or the medial septal area increases aggressive behavior under specific conditions (Edwards and Kravitz, 1997; Takahashi et al., 2012). Therefore, different 5-HT receptors appear to play opposite roles in regulating aggression and the social state in mammals as well.

In summary, we used the loser effect in *Drosophila* as a model to investigate the reversion of the depressive behavioral state that develops secondary to distressful social experiences. The identification of 5-HT signals in our study suggests a possibly universal strategy for overcoming the loser mentality in other species. It would also be interesting to evaluate similar strategies in overcoming other depressive states in certain mental disorders, such as depression and post-traumatic stress disorder.

### Limitations of the Study

Our study demonstrated that activating 5-HT neurons or elevating the 5-HT level overcomes the loser effect in *Drosophila*. We further showed that P1 neurons act upstream and 5-HT1B neurons act downstream of 5-HT neurons. However, while the evidence of functional connectivity between these neurons is relatively strong, the evidence of structural connections is not overwhelming. Moreover, because of issues with genetic handles, we were unable to test the involvement of two 5-HT receptors (5-HT2A and 5-HT2B) or possible interactions between these receptors in the reversal process. Additionally, both behavioral data and activity imaging suggested that 5-HT or 5-HT neurons activate 5-HT1B neurons. This is quite intriguing and deserves a separate investigation in the future, likely involving genetics, biochemistry, imaging/electrophysiology, and behavior.

### Resource Availability

#### Lead Contact

Further information and requests for resources should be directed to and will be fulfilled by the Lead Contact, Yan Zhu (zhuyan@ibp.ac.cn).

#### Materials Availability

All reagents used in this study will be made available on request to the Lead Contact.

#### Data and Code Availability

The original/source data are available from the lead contact on request.

## Methods

All methods can be found in the accompanying Transparent Methods supplemental file.

## Supplemental Information

Document S1. Transparent Methods, Figures S1–S8 and Table S1
