# Supplementary material for: Serotonin Signals Overcome Loser Mentality in Drosophila
Source: iScience. 2020 Oct 6;23(11):101651. doi: 10.1016/j.isci.2020.101651 (PMC7581928; doi:10.1016/j.isci.2020.101651)
Supplement: Document S1. Transparent Methods, Figures S1–S8 and Table S1 [file mmc1.pdf]

iScience, Volume *23*

# Supplemental Information

# Serotonin Signals Overcome

# Loser Mentality in *Drosophila*

**Shao Wei Hu, Yan Tong Yang, Yuanjie Sun, Yin Peng Zhan, and Yan Zhu**

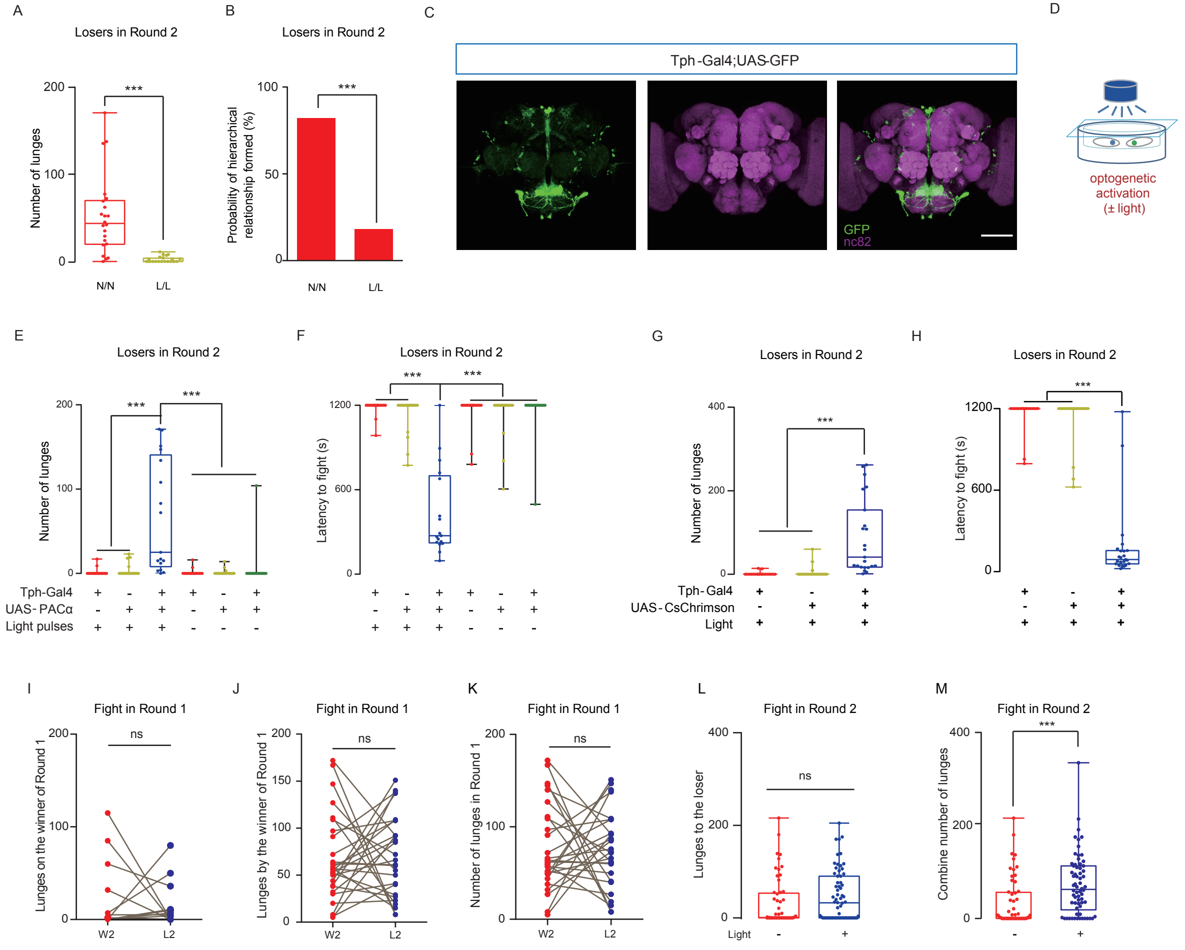
A
Losers in Round 2
Number of lunges
***
N/N
L/L
B
Losers in Round 2
Probability of hierarchical relationship formed (%)
***
N/N
L/L
C
Tph-Gal4;UAS-GFP
GFP
nc82
D
optogenetic activation (± light)
E
Losers in Round 2
Number of lunges
Tph-Gal4
UAS-PACα
Light pulses
F
Losers in Round 2
Latency to fight (s)
G
Losers in Round 2
Number of lunges
Tph-Gal4
UAS-CsChrimson
Light
H
Losers in Round 2
Latency to fight (s)
I
Fight in Round 1
Lunges on the winner of Round 1
ns
W2
L2
J
Fight in Round 1
Lunges by the winner of Round 1
ns
W2
L2
K
Fight in Round 1
Number of lunges in Round 1
ns
W2
L2
L
Fight in Round 2
Lunges to the loser
ns
Light
M
Fight in Round 2
Combine number of lunges
***

**Figure S1. Loser Effect in *Drosophila,* Related to Figures 1 and 2.** (A) Fighting of loser–loser pairs (L/L) in Round 2 exhibited significantly fewer attacks than fighting of naïve–naïve pairs (N/N) in Round 1 (n = 22). (B) Fewer loser pairs established clear winner–loser relationships in Round 2 than did naïve pairs in Round 1 (n = 22). (C) Expression patterns of Tph-Gal4/UAS-mCD8::GFP. (D) Schema of optogenetic stimulation of the losers prior to a fight. (E, F) Optogenetic activation of 5-HT neurons via light pulses (10 Hz) for 80 s increased the attack intensity (E) and decreased the latency to fight (F) in Tph>PACα losers (n = 17–20). (G, H) Optogenetic activation of 5-HT neurons via CsChrimson increased the attack intensity (G) and decreased the latency to fight (H) in losers (n = 20–23). (I-K) In light-activated Tph>PACα flies, the new winner (W2) and loser (L2) in Round 2 were not determined by the numbers of attacks on their opponents (I), by the numbers of attacks from their opponents (J), or by the combined fighting intensity in Round 1 (K) (n = 27). (L, M) Optogenetic activation of 5-HT neurons in losers did not alter the attack actions from the winners (L) or the combined attacks of both winners and losers (M) (n = 57 and 66). All genotypes and experimental conditions are indicated with the plots. In the box-and-whisker plot, the whiskers mark the minimum and maximum, the box includes the 25th to 75th percentiles, and the line within the box indicates the median of the data set. The Mann–Whitney test was performed for (A) and (L)-(M), the Kruskal–Wallis test was performed for (E)-(H), Fisher's exact test was performed for (B), and the paired t-test was performed for (I)-(K). ns, not significant ($P > 0.05$); ***$P < 0.001$. Scale bar, 100 μm.

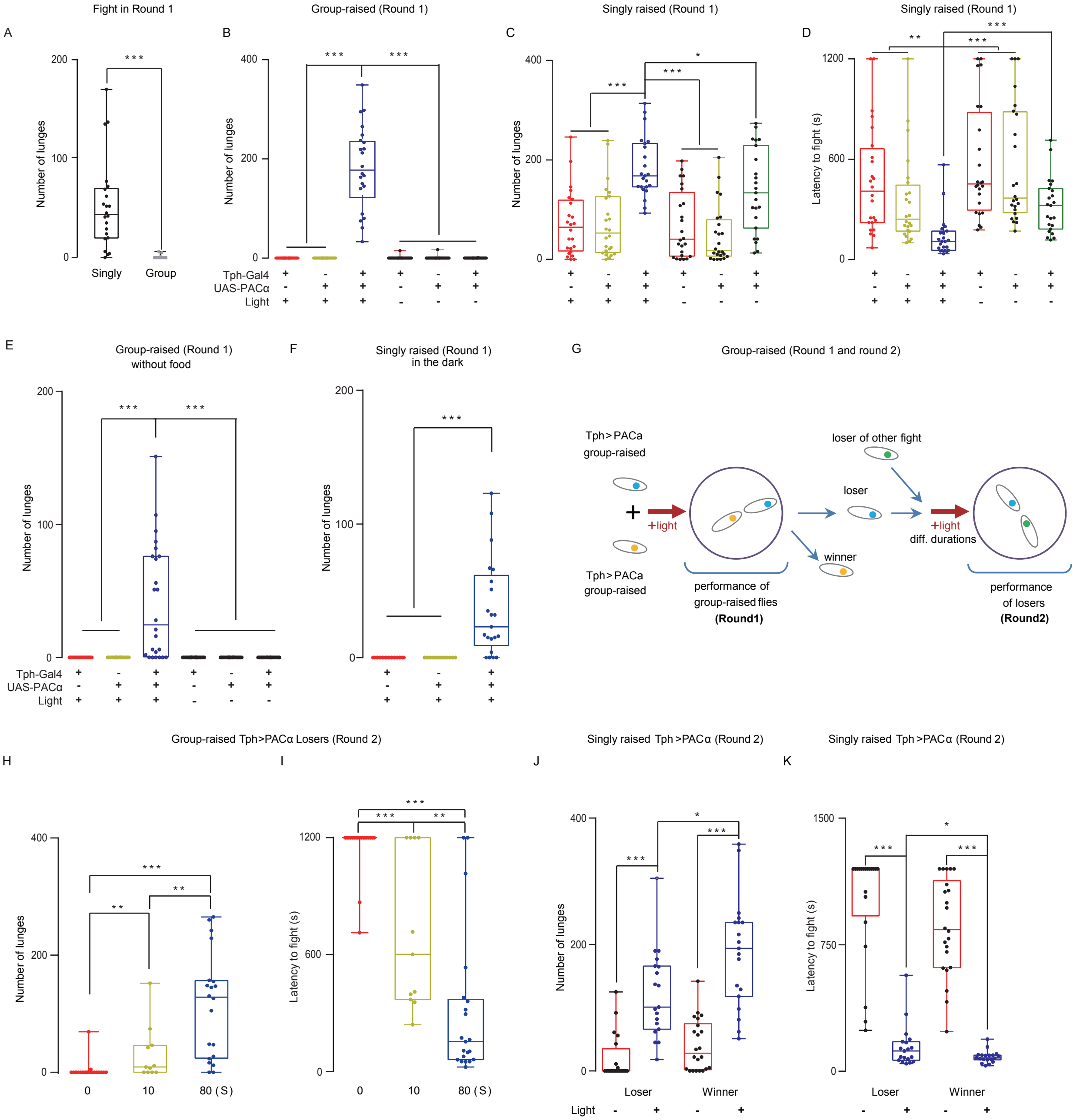

Fight in Round 1
Group-raised (Round 1)
Singly raised (Round 1)
Singly raised (Round 1)
A
B
C
D
Number of lunges
Singly
Group
Tph-Gal4
UAS-PACα
Light
Latency to fight (s)
E
Group-raised (Round 1)
without food
F
Singly raised (Round 1)
in the dark
G
Group-raised (Round 1 and round 2)
Tph>PACa
group-raised
+light
loser of other fight
loser
winner
diff. durations
performance of
group-raised flies
(Round1)
performance
of losers
(Round2)
Group-raised Tph>PACα Losers (Round 2)
Singly raised Tph >PACα (Round 2)
Singly raised Tph >PACα (Round 2)
H
I
J
K
0
10
80 (S)
Loser
Winner
Light

**Figure S2. Activation of 5-HT Neurons Generally Increases Aggression, Related to Figure 2.** (A) Performance of wild-type flies in Round 1. The singly raised males displayed aggressive behavior in contrast to the group-raised males (n = 20–22). (B) Optogenetic activation of Tph-Gal4 neurons induced aggression in the group-raised males (n = 22–24). (C, D) Optogenetic activation of Tph-Gal4 neurons further increased the attack intensity (C) and reduced the latency to fight (D) of socially isolated males (n = 23–24). (E) Optogenetic activation of Tph-Gal4 neurons induced aggression without the presence of food (n = 21–24). (F) Optogenetic activation of Tph-Gal4 neurons induced aggressive actions in the dark (n = 21). (G) Schema of the experimental procedure for testing the loser effect in group-raised Tph>PACα males for (H) and (I). Optogenetic activation of Tph-Gal4 neurons increased attack intensity (H) and decreased the latency to fight (I) in group-raised losers (n = 11–20). (J, K) Optogenetic activation of Tph-Gal4 neurons increased the attack intensity (J) and decreased the latency to fight (K) in winners after 45 minutes of rest (n = 19–22). All genotypes and experimental conditions are indicated with the plots. In the box-and-whisker plot, the whiskers mark the minimum and maximum, the box includes the 25th to 75th percentiles, and the line within the box indicates the median of the data set. The Mann–Whitney test was performed for (A); the Kruskal–Wallis test was performed for (B)-(F) and (H)-(K); $^{*}P < 0.05$; $^{**}P < 0.01$; $^{***}P < 0.001$.

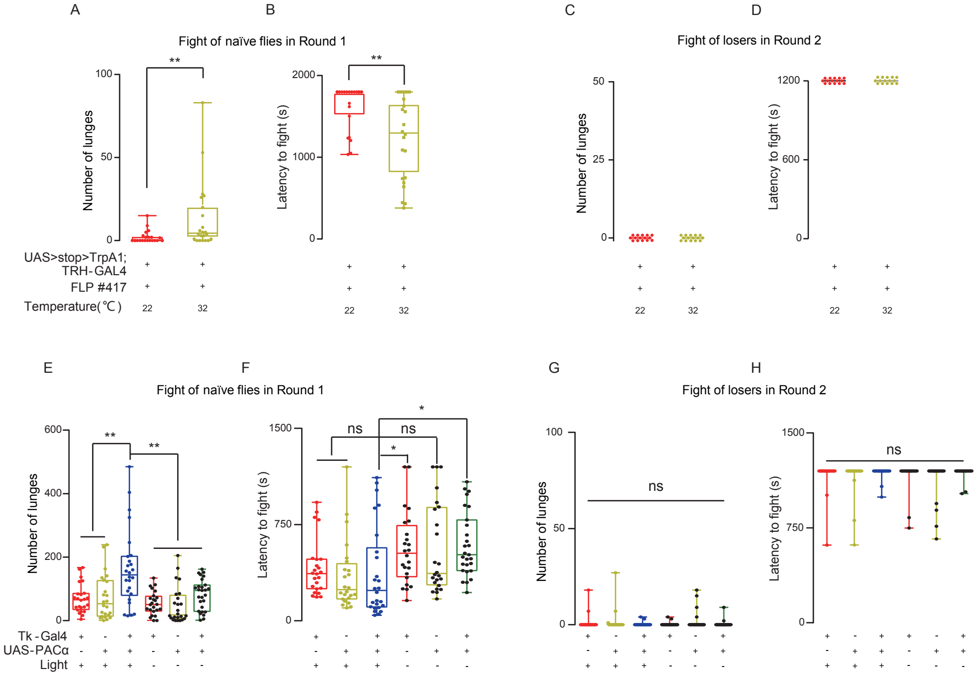

A
B
Fight of naïve flies in Round 1
Number of lunges
Latency to fight (s)
UAS>stop>TrpA1;
TRH-GAL4
FLP #417
Temperature(℃)
22
32
C
D
Fight of losers in Round 2
E
F
Fight of naïve flies in Round 1
G
H
Fight of losers in Round 2
Tk-Gal4
UAS-PACα
Light
ns

**Figure S3. Activation of PLP Neurons and Tk-Gal4 Neurons Is Not Sufficient for Overcoming the Loser Effect, Related to Figure 2.** (A, B) Thermal activation of serotonergic PLP neurons increased the attack intensity (A) and decreased the latency to fight (B) of socially isolated males in Round 1 (n = 22–23). (C, D) Thermal activation of serotonergic PLP neurons did not affect the attack intensity (C) or change the latency to fight (D) of losers in Round 2 (n = 15). (E, F) Photoactivation of Tk-Gal4 neurons increased the attack intensity (E) but did not affect the latency to fight (F) of socially isolated males in Round 1 (n = 24–27). (G, H) Activation of Tk-Gal4 neurons did not affect the attack intensity (G) or change the latency to fight (H) of losers in Round 2 (n = 21–25). All genotypes and experimental conditions are indicated with the plots. In the box-and-whisker plot, the whiskers mark the minimum and maximum, the box includes the 25th to 75th percentiles, and the line within the box indicates median of the data set. The Mann–Whitney test was performed for (A)-(D), the Kruskal–Wallis test was performed for (G)-(H), and one-way ANOVA was performed for (E) and (F). ns, not significant ($P > 0.05$); *$P < 0.05$; **$P < 0.01$.

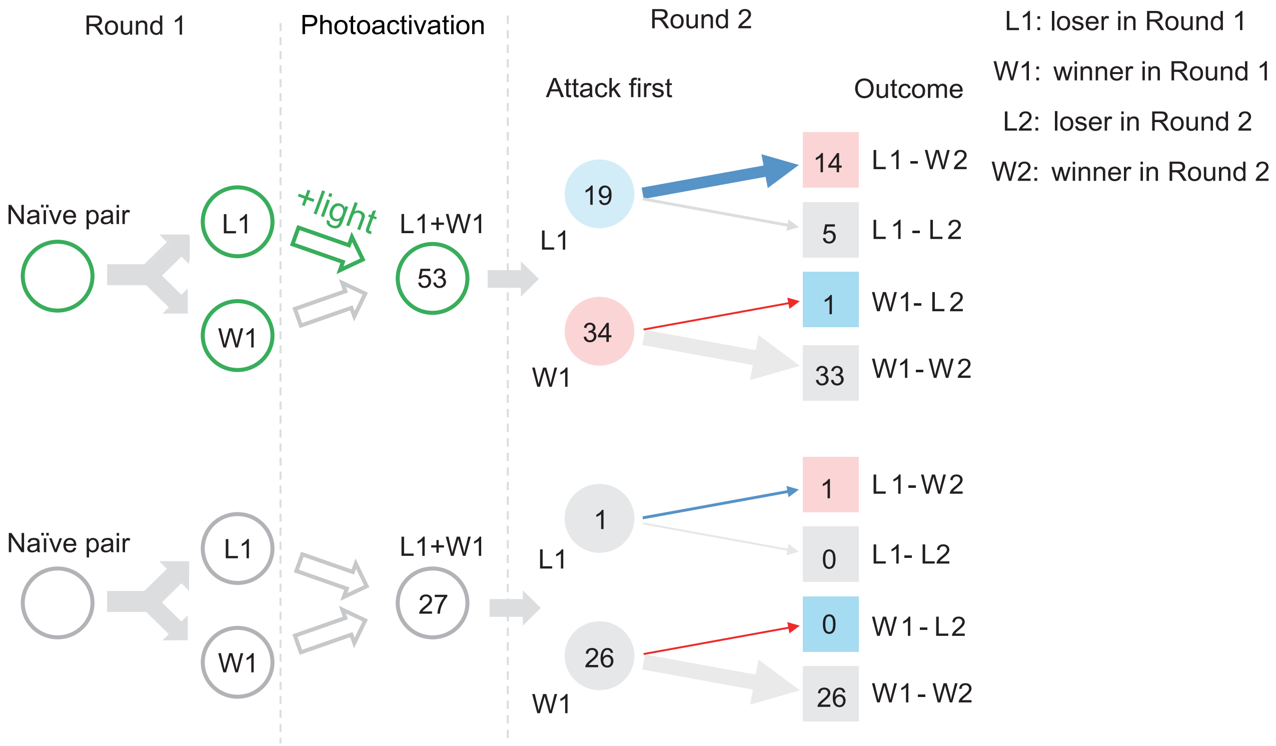

Round 1
Photoactivation
Round 2
Attack first
Outcome
L1: loser in Round 1
W1: winner in Round 1
L2: loser in Round 2
W2: winner in Round 2
Naïve pair
L1
W1
+light
L1+W1
53
19
L1
34
W1
14
L1 - W2
5
L1 - L2
1
W1- L2
33
W1 - W2
Naïve pair
L1
W1
L1+W1
27
1
L1
26
W1
1
L1 - W2
0
L1- L2
0
W1 - L2
26
W1 - W2

**Figure S4. Initiator Effect Accompanies Reversal of Loser Effect, Related to Figure 2.** Familiar loser–winner pairs (Tph>PACα) from Round 1 were put together for Round 2 after the losers had been light-stimulated. The status of initiators (L1 or W1 attacked first) were recorded and correlated to the fighting results. The flies in the control group (bottom) were not treated with light. L1: losers from Round 1; W1: winners from Round 1; L2: losers in Round 2; W2: winners in Round 2. The numbers inside the circles and boxes are the numbers of indicated flies. The expected events (L1 became L2 and W1 became W2) are shown in gray, while the unexpected events (L1 became W2 and W1 became L2) are shown in red (winning) or blue (losing). The thickness of the arrow lines indicates the relative frequency of the indicated events.

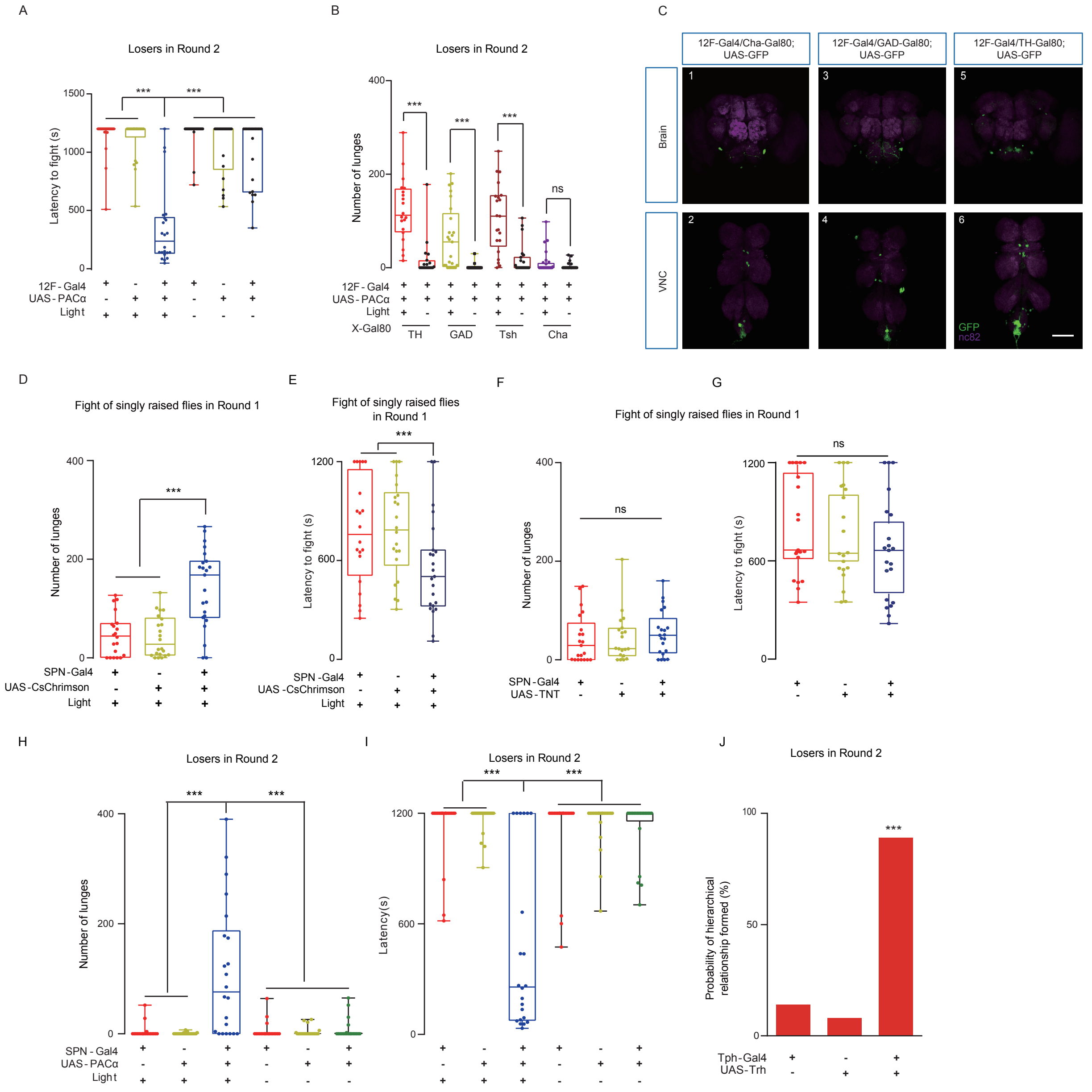

A
Losers in Round 2
Latency to fight (s)
12F-Gal4
UAS-PACα
Light
B
Losers in Round 2
Number of lunges
X-Gal80
TH
GAD
Tsh
Cha
ns
C
12F-Gal4/Cha-Gal80; UAS-GFP
12F-Gal4/GAD-Gal80; UAS-GFP
12F-Gal4/TH-Gal80; UAS-GFP
Brain
VNC
GFP
nc82
D
Fight of singly raised flies in Round 1
Number of lunges
SPN-Gal4
UAS-CsChrimson
Light
E
Fight of singly raised flies in Round 1
Latency to fight (s)
F
Fight of singly raised flies in Round 1
UAS-TNT
G
H
Losers in Round 2
SPN-Gal4
UAS-PACα
I
Losers in Round 2
Latency(s)
J
Losers in Round 2
Probability of hierarchical relationship formed (%)
Tph-Gal4
UAS-Trh

**Figure S5. Enhancing Serotonergic Signals Effectively Extinguishes the Loser Effect, Related to Figure 3.** (A) Photoactivation of 12F-Gal4-labeled 5-HT neurons decreased the latency to fight in losers (n = 22–24). (B) Attack intensities of flies with 12F-Gal4 driving UAS-PACα in the presence of different Gal80s (n = 20–30). (C) Expression patterns of 12F-Gal4 driving UAS-GFP in the presence of Cha-Gal80 (C1 and C2), Gad-Gal80 (C3 and C4), and Th-Gal80 (C5 and C6). (D, E) Optogenetic activation of SPN-split neurons increased the attack intensity (D) and decreased the latency to fight (E) in singly raised males (n = 21–23). (F, G) Silencing SPN-split neurons did not affect the attack intensity (F) or the latency to fight (G) in singly raised males (n = 20–21). (H, I) Optogenetic activation of SPN-split neurons via PACα increased the attack intensity (H) and decreased the latency to fight (I) of losers (n = 21–23). (J) More loser–loser pairs reached a clear winner–loser status than controls when elevating the 5-HT levels in 5-HT neurons (n = 21–28). All genotypes and experimental conditions are indicated with the plots. In the box-and-whisker plot, the whiskers mark the minimum and maximum, the box includes the 25th to 75th percentiles, and the line within the box indicates median of the data set. The Kruskal–Wallis test was performed for (A), (B), (H), and (I); one-way ANOVA was performed for (D)-(G); and the chi-square test was performed for (J) (two-tailed $\chi 2$ = 174.8, df = 2). ns, not significant ($P > 0.05$); ***$P < 0.001$. Scale bar, 100 μm.

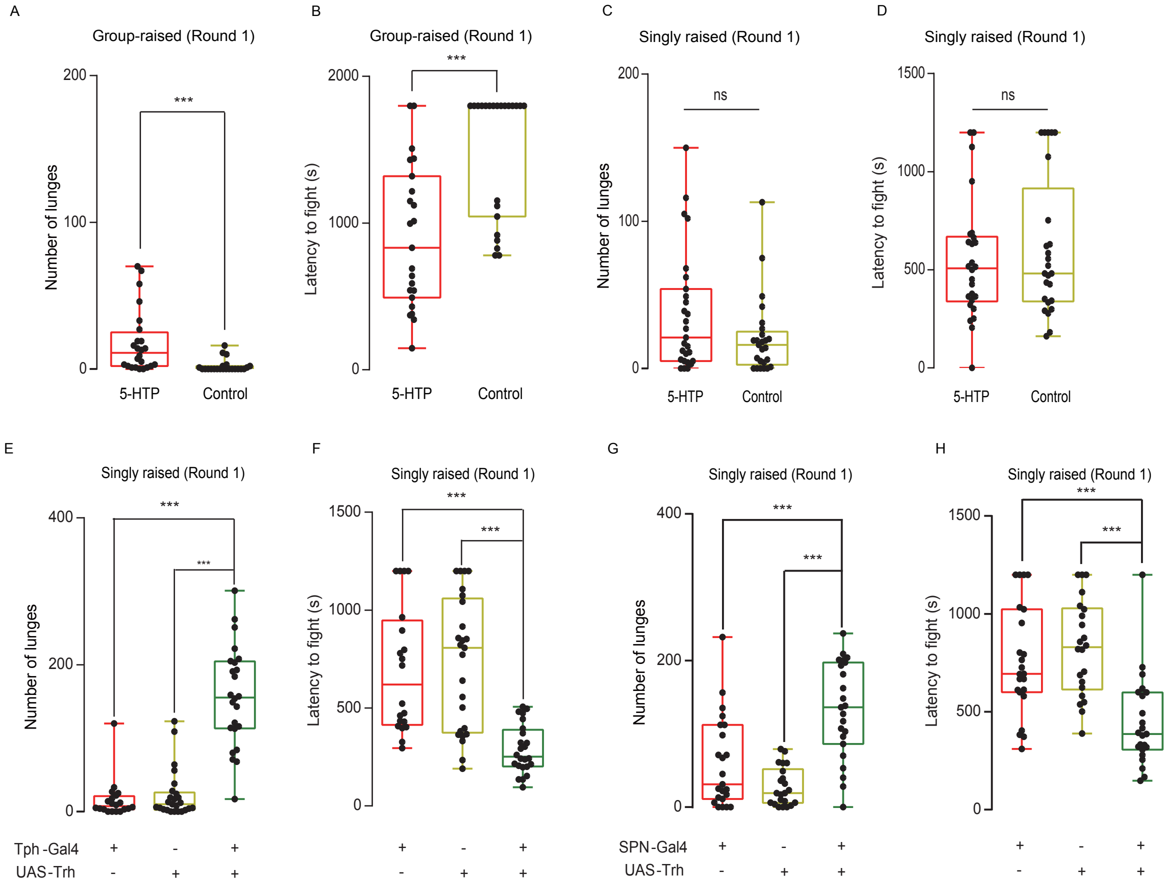

A
Group-raised (Round 1)
Number of lunges
200
100
0
***
5-HTP
Control
B
Group-raised (Round 1)
Latency to fight (s)
2000
1000
0
***
5-HTP
Control
C
Singly raised (Round 1)
Number of lunges
200
100
0
ns
5-HTP
Control
D
Singly raised (Round 1)
Latency to fight (s)
1500
1000
500
0
ns
5-HTP
Control
E
Singly raised (Round 1)
Number of lunges
400
200
0
***
***
Tph -Gal4 + - +
UAS-Trh - + +
F
Singly raised (Round 1)
Latency to fight (s)
1500
1000
500
0
***
***
+ - +
- + +
G
Singly raised (Round 1)
Number of lunges
400
200
0
***
***
SPN-Gal4 + - +
UAS-Trh - + +
H
Singly raised (Round 1)
Latency to fight (s)
1500
1000
500
0
***
***
+ - +
- + +

**Figure S6. Enhanced 5-HT Level Increased Initial Levels of Aggression, Related to Figure 3.** (A, B) Pharmacologically increasing the 5-HT level promoted lunges (A) and decreased latency to fight (B) in group-raised males (n = 23–24). (C, D) Pharmacologically increasing the 5-HT level did not affect the lunge frequency (C) or latency to fight (D) in singly raised males (n = 25–26). (E) Overexpression of 5-HT in Tph neurons increased the lunge frequency in singly raised males (n = 20–25). (F) Overexpression of 5-HT in Tph neurons decreased the latency to fight of singly raised males (n = 20–25). (G) Overexpression of 5-HT in SPNs increased the lunge frequency in singly raised males (n = 22–23). (H) Overexpression of 5-HT in SPNs decreased the latency to fight in singly raised males (n = 22–23). All genotypes and experimental conditions are indicated with the plots. In the box-and-whisker plot, the whiskers mark the minimum and maximum, the box includes the 25th to 75th percentiles, and the line within the box indicates median of the data set. The Mann–Whitney test was performed for (A) and (B), the t-test was performed for (C) and (D), and the Kruskal–Wallis test was performed for (E)-(H). ns, not significant ($P > 0.05$); ***$P < 0.001$.

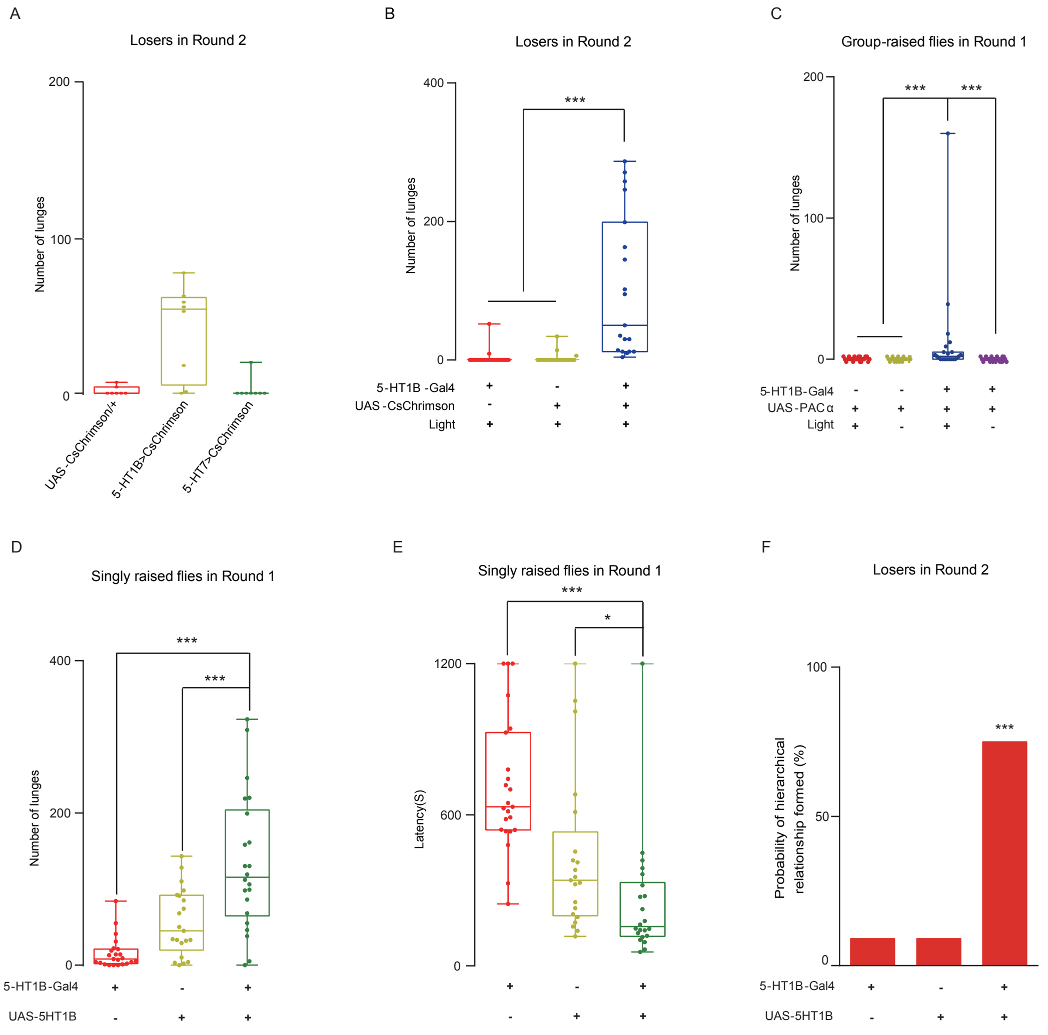

A
Losers in Round 2
Number of lunges
200
100
0
UAS-CsChrimson/+
5-HT1B>CsChrimson
5-HT7>CsChrimson
B
Losers in Round 2
Number of lunges
400
200
0
***
5-HT1B -Gal4 + - +
UAS -CsChrimson - + +
Light + + +
C
Group-raised flies in Round 1
Number of lunges
200
100
0
*** ***
5-HT1B-Gal4 - - + +
UAS-PAC α + + + +
Light + - + -
D
Singly raised flies in Round 1
Number of lunges
400
200
0
***
***
5-HT1B-Gal4 + - +
UAS-5HT1B - + +
E
Singly raised flies in Round 1
Latency(S)
1200
600
0
***
*
+ - +
- + +
F
Losers in Round 2
Probability of hierarchical relationship formed (%)
100
50
0
***
5-HT1B-Gal4 + - +
UAS-5HT1B - + +

**Figure S7. 5-HT1B-Positive Neurons Control Aggressive Arousal of Losers, Related to Figure 4.** (A) Survey of serotonin receptors for overcoming the loser effect. Neurons of 5-HT receptors in losers were activated optogenetically, and the numbers of attacks of loser pairs in Round 2 were quantified (n = 8). (B) Optogenetic activation of 5-HT1B receptor neurons via CsChrimson promoted aggression in losers (n = 19–23). (C) Activation of 5-HT1B neurons induced aggression in the group-raised males in Round 1 (n = 23–24). (D) Increased 5-HT1B level in 5-HT1B neurons elevated aggression in the singly raised males (n = 21–23). (E) Increased 5-HT1B level in 5-HT1B neurons reduced the latency to fight of singly raised males (n = 21–23). (F) More loser–loser pairs reached a clear winner–loser status when 5-HT1B levels were elevated in 5-HT1B neurons (n = 22–24). All genotypes and experimental conditions are indicated with the plots. In the box-and-whisker plot, the whiskers mark the minimum and maximum, the box includes the 25th to 75th percentiles, and the line within the box indicates median of the data set. The Kruskal–Wallis test was used for (B)-(E), and the chi-square test was used for (F) (two-tailed $\chi 2 = 135.8$, df = 2). *$P < 0.05$; ***$P < 0.001$.

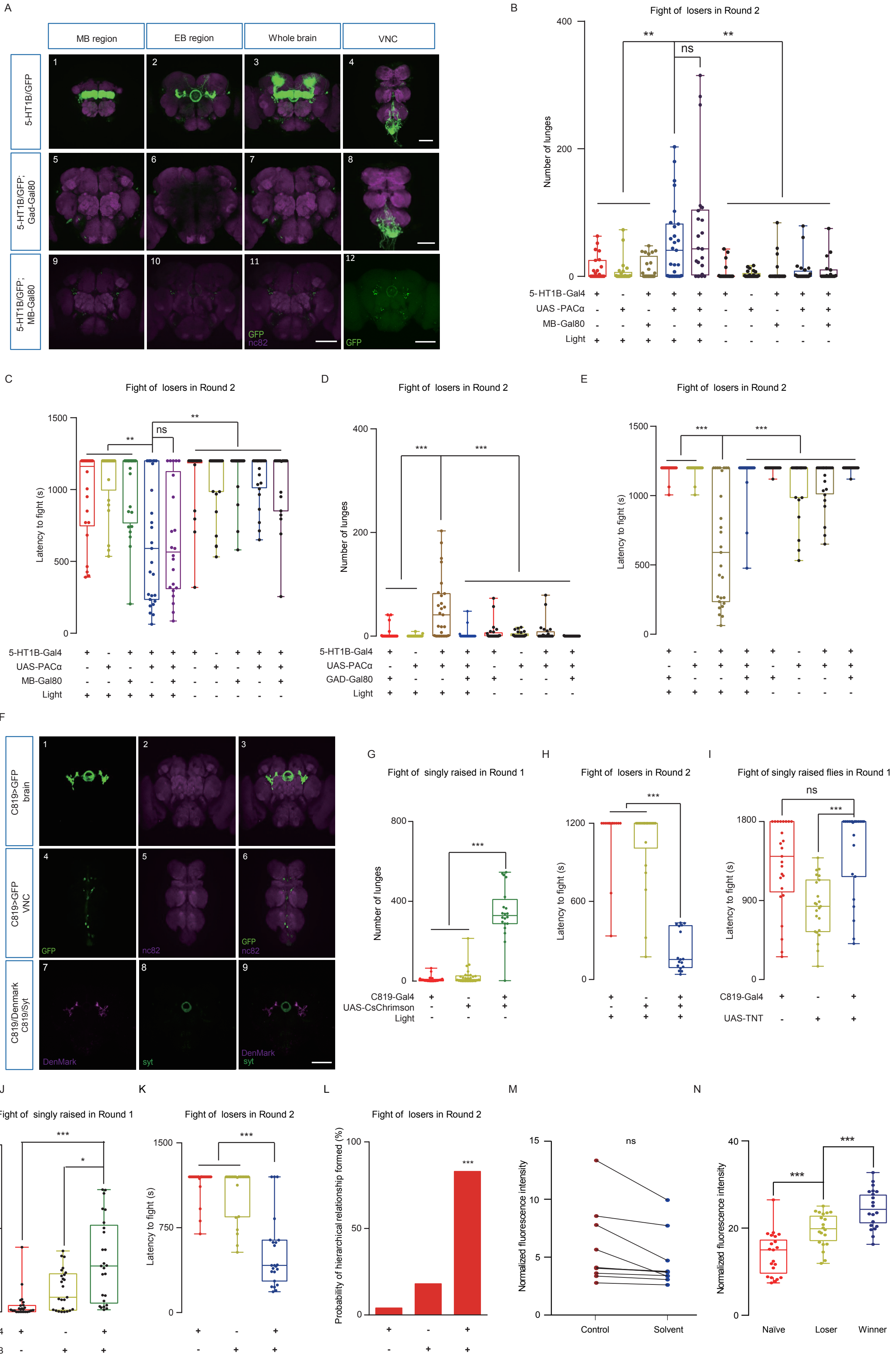

A
MB region
EB region
Whole brain
VNC
5-HT1B/GFP
5-HT1B/GFP; Gad-Gal80
5-HT1B/GFP; MB-Gal80
GFP
nc82
B
Fight of losers in Round 2
Number of lunges
5-HT1B-Gal4
UAS -PACα
MB-Gal80
Light
C
Fight of losers in Round 2
Latency to fight (s)
5-HT1B-Gal4
UAS-PACα
MB-Gal80
Light
D
Fight of losers in Round 2
Number of lunges
5-HT1B-Gal4
UAS-PACα
GAD-Gal80
Light
E
Fight of losers in Round 2
Latency to fight (s)
F
C819>GFP brain
C819>GFP VNC
C819/Denmark C819/Syt
GFP
nc82
DenMark
syt
G
Fight of singly raised in Round 1
Number of lunges
C819-Gal4
UAS-CsChrimson
Light
H
Fight of losers in Round 2
Latency to fight (s)
I
Fight of singly raised flies in Round 1
Latency to fight (s)
C819-Gal4
UAS-TNT
J
Fight of singly raised in Round 1
Number of lunges
C819-Gal4
UAS-5HT1B
K
Fight of losers in Round 2
Latency to fight (s)
L
Fight of losers in Round 2
Probability of hierarchical relationship formed (%)
M
Normalized fluorescence intensity
Control
Solvent
N
Normalized fluorescence intensity
Naïve
Loser
Winner

**Figure S8. 5-HT1B-Positive C819 Neurons in EB Control Aggressive Arousal of Losers, Related to Figure 5.** (A) Expression patterns in males of 5-HT1B-Gal4/UAS-mCD8::GFP (A1-A4), 5-HT1B-GAL4/ UAS-mCD8::GFP; Gad-Gal80 (A5-A8), and 5-HT1B-GAL4/ UAS-mCD8::GFP; MB-Gal80 (A9-A11) in the MB, ellipsoid body, and whole brain. GFP signals in A12 were enhanced specifically from A11 to show the remaining neurons. (B, C) Attack intensity (B) and latency to fight (C) remained unchanged in loser pairs of 5-HT1B-Gal4 driving UAS-PACα with or without MB-Gal80, which prevents optogenetic activation of MB neurons (n = 19–27). (D, E) The attack intensity was decreased (D) and the latency to fight was increased (E) in loser pairs of 5-HT1B-Gal4 driving UAS-PACα with Gad-Gal80 (n = 23–27). (F) Expression patterns of C819-Gal4/UAS-mCD8::GFP in the brain (F1-F3) and VNC (F4-F6). F7 shows the postsynaptic patterns of C819 neurons as indicated by C819>DenMark, whereas F8 shows the pre-synaptic patterns of C819 neurons as indicated by C819>Synaptotagmin-GFP (syt). (G) Activation of C819 neurons increased the lunge frequency of singly raised males (n = 20–30). (H) Optogenetic activation of C819 neurons decreased the latency to fight in losers (n = 14–22). (I) Inhibition of synaptic transmission in C819 neurons did not change the latency to fight in socially isolated males (n = 23–25). (J) Elevating expression levels of the 5-HT1B receptor in C819 neurons increased lunge frequency of singly raised males (n = 25). (K) Elevating expression levels of the 5-HT1B receptor in C819 neurons decreased the latency to fight in losers (n = 22–24). (L) More loser–loser pairs formed stable winner–loser relationships when the 5-HT1B level was elevated in C819 neurons (n = 22–24). (M) Application of solvent alone, instead of 5-HT, did not increase the activity of C819 neurons (n = 10). (N) The activity of R2/R4m neurons in both winners and losers was higher than that in naïve flies (n = 20–21). All genotypes and experimental conditions are indicated with the plots. In the box-and-whisker plot, the whiskers mark the minimum and maximum, the box includes the 25th to 75th percentiles, and the line in the box indicates the median of the data set. The Kruskal–Wallis test was performed for (B)-(E), (G)-(K). The chi-square test was performed for (L) (two-tailed $\chi 2 = 156.2$, df = 2), the paired t-test was performed for (M), and one-way ANOVA was performed for (N). ns, not significant ($P > 0.05$); *$P < 0.05$, **$P < 0.01$, and ***$P < 0.001$. Scale bar, 100 μm.

**Supplementary Table S1. *Drosophila* strains, reagents, software, and source data essential to reproduce results presented in the manuscript, Related to Figures 1–8 and Transparent Methods.**

| REAGENT or RESOURCE | SOURCE | IDENTIFIER |
|---|---|---|
| **Antibodies** | | |
| anti-GFP (rabbit) | Thermo Fisher | CAT# A-6455<br>RRID AB_221570 |
| fasciclin II (mouse) | DSHB | CAT# ab141801<br>RRID AB_28235 |
| nc82 (mouse) | DSHB | CAT# ZF-0313<br>RRID AB_2314866 |
| Goat anti-Rabbit IgG (H+L) Alexa Fluor 488 | ZSGB-Bio | CAT# ZF-0511 |
| Goat Anti-Mouse IgG (H+L) Alexa Fluor 635 | Thermo Fisher | CAT# A31574<br>RRID AB_2536185 |
| **Chemicals, Peptides, and Recombinant Proteins** | | |
| 5-Hydroxy-L-tryptophan | Sigma-Aldrich | CAT# H9772-1G |
| Serotonin hydrochloride | Alfa | CAT# B21263 |
| Adenosine 5'-triphosphate disodium salt hydrate | Sigma | CAT# A2383-5G |
| **Experimental Models: Organisms/Strains** | | |
| *Drosophila: Wild type Canton S* | Li Liu | N/A |
| *Drosophila: Tph-Gal4* | Yi Rao | Park et al. 2006 |
| *Drosophila: UAS-PACα* | B. Kottler and M. Schwarzel. | N/A |
| *Drosophila: UAS-NaChBac (TM6B)* | Bloomington Drosophila Stock Center | RRID: BDSC_9468 |
| *Drosophila: UAS>stop>TrpA1;TRH-Gal4* | Olga V. Alekseyenko, Edward A. Kravitz | N/A |
| *Drosophila: FLP # 417* | Olga V. Alekseyenko, Edward A. Kravitz | N/A |
| *Drosophila: Tk-GAL4* | Bloomington Drosophila Stock Center | RRID: BDSC_51975 |
| *Drosophila: 12F-Gal4* | Olga V. Alekseyenko, Edward A. Kravitz | N/A |
| *Drosophila: UAS-mCD8::GFP* | Y. Li and A. Guo | N/A |

| | | |
|---|---|---|
| *Drosophila: Trh-Gal4* (second chromosome) | Olga V. Alekseyenko, Edward A. Kravitz | N/A |
| *Drosophila: Trh-Gal4* (third chromosome) | Olga V. Alekseyenko, Edward A. Kravitz | N/A |
| *Drosophila: UAS-Trh* | Bloomington Drosophila Stock Center | RRID: BDSC_27638 |
| *Drosophila: UAS-Trh-RNAi* | Julie H. Simpson | Albin et al., 2015 |
| *Drosophila: Th-Gal80* | Y. Li and A. Guo | N/A |
| *Drosophila: Cha-Gal80* | Y. Li and A. Guo | N/A |
| *Drosophila: Gad-Gal80* | Y. Li and A. Guo | N/A |
| *Drosophila: MB-GaL80* | Y. Li and A. Guo | N/A |
| *Drosophila: SL3 (5-HT1B-Gal4)* | Yi Rao | Quan et al. 2005 |
| *Drosophila: HMS6 (5-HT1B-Gal4)* | Yi Rao | Quan et al. 2005 |
| *Drosophila: HMS1 (UAS-5-HT1B)* | Yi Rao | Quan et al. 2005 |
| *Drosophila: UAS-TNT* | Y. Li and A. Guo | N/A |
| *Drosophila: 20XUAS-IVS-CsChrimson.mVenus (attP40)* | Bloomington Drosophila Stock Center | RRID: BDSC_5515 |
| *Drosophila: 13XLexAop2-IVS CsChrimson.mVenus (attP2)* | Bloomington Drosophila Stock Center | RRID: BDSC_5519 |
| *Drosophila: 5-HT2A-Gal4* | Yi Rao | N/A |
| *Drosophila: 5-HT2B-Gal4* | Yi Rao | N/A |
| *Drosophila: 5-HT7-Gal4* | Yi Rao | N/A |
| *Drosophila: tsh-GaL80* | L. Vosshall | N/A |
| *Drosophila: C819-Gal4* | Li Liu | N/A |
| *Drosophila: UAS-Denmark* | Y. Li and A. Guo | N/A |
| *Drosophila: UAS-Synaptotagmin-GFP* | Y. Li and A. Guo | N/A |
| *Drosophila: UAS-RFP* | Bloomington Drosophila Stock Center | RRID: BDSC_31417 |
| *Drosophila: GMR15A01-lexA (attp40)* | Bloomington Drosophila Stock Center | RRID: BDSC_54426 |
| *Drosophila: GMR15A01-Gal4 (attp2)* | Bloomington Drosophila Stock Center | RRID: BDSC_48670 |
| *Drosophila: 20XUAS-IVS-GCaMP6m (attP40)* | Bloomington Drosophila Stock Center | RRID: BDSC_42748 |
| *Drosophila: LexAop-P2X$_2$;UAS-GCamp6m* | Yufeng Pan | N/A |

| | | |
|---|---|---|
| *Drosophila*: *UAS-myrGFP.QUAS-mtdTomato-3xHA (attP8); trans-Tango (attP40)* | Bloomington Drosophila Stock Center | RRID: BDSC_77124 |
| *Drosophila*: *LexAop-spGFP11; UAS-spGFP1-10* | Mark Wu | N/A |
| *Drosophila*: *SPN split-Gal4* | Thomas Preat | N/A |
| **Software and Algorithms** | | |
| Prism6 | GraphPad Software | URL: http://www.graphpad.com/; RRID:SCR_002798 |
| MATLAB R2012a | MathWorks | URL: http://www.mathworks.com/products/matlab/; RRID:SCR_001622 |

**Transparent Methods**

**Fly stocks and genetics.** Flies were usually reared at 25°C and 60% humidity in a 12:12-h light:dark regimen (light on at 08:00) unless otherwise indicated. The standard fly media included water (1000 mL), cornmeal (77.7 g), yeast (32.1 g), agar (8 g), calcium chloride (0.726 g), sucrose (31.62 g), glucose (63.2 g), potassium sorbate (2 g), and methyl p-hydroxybenzoate (1.5 g). The flies used for the optogenetic experiments were reared in the dark. Flies for CsChrimson experiments were raised without all-trans-retinal.

The details of the fly stocks are listed in Supplementary Table S1. Brief descriptions of three lines (CS, 12F-Gal4, and SPNsplit-Gal4) are provided below. The wild-type strain used was Canton S (CS; stock number: 105666; Kyoto Stock Center). Flies with 12F-Gal4 were obtained from O. Alekseyenko and E. Kravitz. The transgene for 12F-Gal4 was first generated by fusing the short regulatory sequence of the neural-specific Trh gene (CG9122) with Gal4; it was then inserted into the second chromosome (Alekseyenko et al., 2010). The SPN split-Gal4 flies were obtained from T. Preat and included two transgenes: an activation domain fused with a regulatory sequence from VT026326 (inserted into the attp40 site) and a DNA-binding domain fused with a regulatory sequence from VT057280 (inserted into the attp2 site) (Scheunemann et al., 2018). Behavioral assays were carried out at 25°C and 60% humidity between 15:00 and 19:00.

**Aggression assay.** The protocol for measuring aggression was adapted from previous work with some modifications (Zhou et al., 2008). The circular fighting chamber had a radius of 7 mm and contained a central food patch (radius of 4 mm). The inside height of the chamber was 3.5 mm. Among the socially isolated flies, newly emerged flies were collected after eclosion and reared individually in a 2-mL Eppendorf tube containing 0.5 mL of food. Flies aged 6 to 8 days were used for behavioral tests. To distinguish the flies from one another, they were lightly anesthetized with carbon dioxide and marked on the thorax with acrylic paint of different colors. Painted flies were allowed to recover for at least 48 h before aggression assays were carried out.

Antagonistic interactions between a pair were quantified by the number of lunges and the latency to fight. The number of lunges was measured by the number of combined lunges performed by both flies within the first 20 min of observation. The latency to fight was measured as the duration of time from the placement of the flies into the chamber to the first aggressive action. To simplify quantification, when no aggressive actions had occurred throughout the 20 min period, the latency to fight was assigned a

1200 s. In a few experiments, the observation period was 30 min. The first fly to lunge was defined as the initiator. The criterion of assigning winners and losers was based on a five-lunge/five-retreat rule (Yurkovic et al., 2006).

**Social defeat assay.** The protocol for the social defeat assay was adapted from previous work with some modifications (Yurkovic et al., 2006). For the Round 1 fight, two singly raised males were placed in a standard fighting chamber for 30 min. After 30 min of fighting, most naïve–naïve pairs formed a stable winner–loser relationship. The loser flies were then returned to their home vials for 30 min and paired for 20 min with a loser or winner for Round 2. The assignment of winners and losers was based on a five-lunge/five-retreat criterion. We quantified the fighting intensity with the combined number of lunges and latency of fighting (the elapsed time until the first lunge was performed).

**State persistence assay.** After naïve–naïve pairs of Tph>PACα males formed stable winner–loser relationships, we removed the winners, illuminated the losers inside the fighting chambers for 80 s, and returned the losers to their home tubes for 15, 30, or 60 min. We then tested the aggression of these losers.

**Territory assay.** The protocol for the territory assay was adapted from previous work with some modifications (Zhou et al., 2008). A Tph>PACα male and Tph>NaChBac male were paired in a fighting chamber. When a Tph>PACα male became a loser, we illuminated the flies with blue light for 80 s to activate the 5-HT neurons of the Tph>PACα loser. The duration of time that each fly stayed on the food patch of the fighting chamber within the first 5 min was quantified.

**Threat display assay.** A fly (the threatener) displays threat-like actions prior to engaging in physical contact for a fight (Duistermars et al., 2018). We quantified the threat displays of a threatener by analyze the number of wing elevation and charges toward the opponent in the first 5 min (0-5 min) after placing them in a fighting chamber.

**Courtship motivation assay.** The analysis for courtship motivation was adapted from previous work with some modifications (Teseo et al., 2016). We used decapitated females, which usually stood and exhibited basic activities for several hours but did not respond to courtship attempts from males, thus allowing us to quantify the males' motivation to mate objectively. In contrast, intact females avoid, reject, or mate with males, influencing the males' behavior and thus increasing the complexity of data analysis.

After 30 min of fighting, the winners and losers were separated and returned to their home vials for 30 min. We then individually tested the courtship attempts of winners, losers, and singly raised naïve flies toward decapitated virgin females for 20 min each.

**Optogenetic stimulation.** For continuous optogenetic stimulation, a 460-nm blue light source (Denjoy DY400-4) was placed 5 mm above the fighting chambers to illuminate the flies inside for 80 s before starting video recording. The light intensity (122 $mW/cm^2$) was measured with a spectrometer (CCS200/M, Thorlabs) at the site of illumination. The fighting chambers were topped with a transparent glass sheet for illumination and observation.

The intensity and duration of light illumination were determined by pilot experiments. Half of the minimal intensity causing paralysis in elav>PACα flies was chosen for optogenetic manipulation. At this intensity, the exposure time (80 s) was found to induce the highest number of lunges in group-raised Tph>PACα flies. Under this condition, the behavioral effects induced by PACα and CsChrimson were similar with most drivers.

To generate light pulses for optogenetic activation, a 480-nm square LED of 20 × 20 mm (RJH100B160A1-1500T; Ruijiahong) was controlled by an Arduino board with a custom script to deliver 10-Hz pulses (pulse width of 20 ms) to a fighting chamber at a 45-degree angle. The illumination duration and light intensity for photoactivation experiments were the same as described above.

**Thermogenetic activation.** Activating the 5HT-PLP neurons required thermogenetic manipulation. The protocol was adapted from previous work with some modifications (Alekseyenko et al., 2014). Newly emerged flies were collected after eclosion and maintained at 18°C for 10 to 12 days before testing. The dTrpA1 activation in Round 1 was conducted at 32°C to increase the efficacy of dTrpA1 activation in the experimental groups, whereas the temperature-controlled groups were tested at 22°C. Losers were generated by allowing a pair to fight at 22°C for 30 min. The losers were then tested at 32°C to evaluate their fighting behavior with the losers fought at 22°C, serving as the temperature controls.

**Drug treatment.** The pharmacological protocol was adapted from previous work with some modifications (Dierick and Greenspan, 2007). Newly emerged males were

collected after eclosion and reared individually in a 2-mL Eppendorf tube containing 0.5 mL of food for 3 days, then transferred into a new 2-mL Eppendorf tube containing 0.5 mL of food mixed with 5-HTP (H9772; Sigma) at a final concentration of 50 mM for 3 days.

**Immunohistochemistry.** The immunohistochemistry protocol was adapted from previous work with some modifications (Zhan et al., 2016). Dissection of intact brains and VNCs of adult male flies was performed in cold phosphate-buffered saline and fixed in 4% fresh paraformaldehyde solution for 3 to 4 h on ice. The tissues were then washed with 0.1% Triton X-100 in 1× phosphate-buffered saline (PBT) three times (15 min each), blocked for 30 min with PBT containing 5% normal goat serum, and incubated with a primary antibody in a blocking buffer for 24 h at 4°C. After washing with PBT three times, the tissues were incubated with a secondary antibody in PBT for 24 h at 4°C. The nc82 signals served as counterstaining unless otherwise indicated. We used the same protocol as above for *trans*-Tango imaging. The flies for *trans*-Tango analysis were raised at 18°C for 10 to 20 days before dissection.

**Functional fluorescence imaging.** Previously established methods for calcium imaging were used with minor modifications (Chen et al., 2017). Adult hemolymph-like saline (AHL) consisting of 108 mM NaCl, 5 mM KCl, 2 mM $CaCl_2$, 8.2 mM $MgCl_2$, 4 mM $NaHCO_3$, 1 mM $NaH_2PO_4$-$H_2O$, 5 mM trehalose, 10 mM sucrose, and 5 mM HEPES (pH 7.5) was used to bathe the brains for dissection and imaging. Flies were briefly anesthetized on ice, and the brains were quickly dissected into AHL at room temperature. For imaging, an O-ring (inner diameter of 10 mm) was glued to a glass slide to form a small reservoir to hold a brain in AHL.

To measure the levels of activity in C819 neurons after fights, two C819>GCaMP6; myr::RFP males were placed in a fighting chamber for 30 to 60 min of fighting. The brains of losers and winners were dissected and imaged *in vitro*. We used C819>GCaMP6; myr::RFP males placed individually in the fighting chamber for 30 to 60 min as the socially isolated controls (socially isolated males treated similarly but singly in a fighting chamber for the same duration).

To measure the changes in the activity of C819 neurons after 5-HT application, whole-brain explants of C819>GCaMP6; myr::RFP were positioned on circular coverslips (5-mm diameter) and placed in a recording chamber containing AHL. Serotonin hydrochloride (1 mM, dissolved in AHL) was gently delivered by a pipette into the chamber. The control group received only AHL.

For the ATP application experiments, whole-brain explants of GMR15A01-LexA/LexAop-P2X2; C819/UAS-GCamp6m (or the control: GMR15A01-lexA/+; C819/UAS-GCamp6m) were placed in a recording chamber containing AHL. ATP dissolved in AHL was gently delivered into the chamber to reach a final ATP concentration of 2.5 mM.

Calcium imaging was performed using an SP8 confocal microscope (Leica, Wetzlar, Germany) with a 20× objective (Figures 5H and 5I) or a 40× water immersion objective (Figures 5F, 5G and Figures 6C-6E). All settings were kept constant between the experimental conditions. Images were taken in 2.0-μm steps and acquired at 512 × 512 pixels. GCaMP fluorescence was measured with excitation at 488 nm. Fluorescence signals were recorded at 0.7 Hz. Images were processed with customized scripts of MATLAB (MathWorks).

RFP signals were used to analyze the data of the activity in C819 neurons (after fights and 5-HT application). First, the raw data were loaded using the Bio-Formats library (Linkert et al., 2010). Next, the three-dimensional region of interest (ROI) of the EB was manually defined according to the RFP signal. Finally, the green fluorescent protein intensities within the ROI were averaged to represent the quantified GCaMP signal.

For the imaging experiments performed to evaluate the functional connectivity between P1 and C819 neurons, the fly of GMR15A01-LexA/LexAop-P2X2; C819/UAS-GCaMP6m did not have an RFP transgene, so the three-dimensional ROIs were built manually with the help of a baseline GFP signal from the GCaMP. With the same three-dimensional ROI for one time series, the GCaMP fluorescent values were then obtained by averaging signals across all the pixels at each time point. The change in fluorescent intensity was calculated as follows: $\Delta F/F0 = (Ft - F0) / F0 \times 100$, where Ft is the fluorescent value at time t, and F0 is the averaged value of three-time points near time zero (before ATP was applied). All imaging and analyses were performed blinded to the experimental conditions.

**Statistical analysis.** Statistical analysis was performed using Prism 6 (GraphPad Software, La Jolla, CA, USA). All experiments were performed in parallel with both the experimental and control groups. All data points in a data set were plotted in a box-and-whisker plot; the whiskers marked the minimum and maximum of the data set, the box included data from the 25th to 75th percentiles, and the line within indicated the median. When two groups of normally distributed data were compared, we performed Student's t-test. The Kruskal–Wallis test was used to analyze

non-normally distributed data. Analysis of variance (ANOVA) was used to analyze multiple comparisons among data with normal distributions. Following ANOVA, the Bonferroni post hoc test was conducted to determine statistical significance.

## Supplemental References

Alekseyenko, O.V., Chan, Y.B., Fernandez, M.D., Bulow, T., Pankratz, M.J., and Kravitz, E.A. (2014). Single Serotonergic Neurons that Modulate Aggression in Drosophila. Curr Biol *24*, 2700-2707.

Alekseyenko, O.V., Lee, C., and Kravitz, E.A. (2010). Targeted manipulation of serotonergic neurotransmission affects the escalation of aggression in adult male Drosophila melanogaster. PLoS One *5*, e10806.

Chen, D.D., Sitaraman, D., Chen, N., Jin, X., Han, C.H., Chen, J., Sun, M.S., Baker, B.S., Nitabach, M.N., and Pan, Y.F. (2017). Genetic and neuronal mechanisms governing the sex-specific interaction between sleep and sexual behaviors in Drosophila. Nat Commun *8*, 154.

Dierick, H.A., and Greenspan, R.J. (2007). Serotonin and neuropeptide F have opposite modulatory effects on fly aggression. Nat Genet *39*, 678-682.

Duistermars, B.J., Pfeiffer, B.D., Hoopfer, E.D., and Anderson, D.J. (2018). A Brain Module for Scalable Control of Complex, Multi-motor Threat Displays. Neuron *100*, 1474-1490.

Linkert, M., Rueden, C.T., Allan, C., Burel, J.M., Moore, W., Patterson, A., Loranger, B., Moore, J., Neves, C., Macdonald, D., *et al.* (2010). Metadata matters: access to image data in the real world. The Journal of cell biology *189*, 777-782.

Scheunemann, L., Plaçais, P.Y., Dromard, Y., Schwärzel, M., and Preat, T. (2018). Dunce Phosphodiesterase Acts as a Checkpoint for Drosophila Long-Term Memory in a Pair of Serotonergic Neurons. Neuron *98*, 350-365.e355.

Teseo, S., Veerus, L., and Mery, F. (2016). Fighting experience affects fruit fly behavior in a mating context. Die Naturwissenschaften *103*, 38.

Yurkovic, A., Wang, O., Basu, A.C., and Kravitz, E.A. (2006). Learning and memory associated with aggression in Drosophila melanogaster. Proc Natl Acad Sci U S A *103*, 17519-17524.

Zhan, Y.P., Liu, L., and Zhu, Y. (2016). Taotie neurons regulate appetite in Drosophila. Nat Commun *7*, 13633.

Zhou, C., Rao, Y., and Rao, Y. (2008). A subset of octopaminergic neurons are important for Drosophila aggression. Nat Neurosci *11*, 1059-1067.
